# Constructing a competitive endogenous RNA network of EndMT-related atherosclerosis through weighted gene co-expression network analysis

**DOI:** 10.3389/fcvm.2023.1322252

**Published:** 2024-01-10

**Authors:** Yawei Li, Yubiao Wu, Xiude Qin, Jinchao Gu, Aijun Liu, Jiahui Cao

**Affiliations:** ^1^ Research Center of Basic Integrative Medicine, School of Basic Medical Sciences, Guangzhou University of Chinese Medicine, Guangzhou, China; ^2^ Encephalopathy Department, Shenzhen Traditional Chinese Medicine Hospital, The Fourth Clinical Medical College of Guangzhou University of Chinese Medicine, Shenzhen, China

**Keywords:** atherosclerosis, endothelial-to-mesenchymal transition, Dan-Shen-Yin, WGCNA, cirRNA

## Abstract

Atherosclerosis is a chronic inflammatory disease characterized by endothelial dysfunction and plaque formation. Under pro-inflammatory conditions, endothelial cells can undergo endothelial-to-mesenchymal transition (EndMT), contributing to atherosclerosis development. However, the specific regulatory mechanisms by which EndMT contributes to atherosclerosis remain unclear and require further investigation. Dan-Shen-Yin (DSY), a traditional Chinese herbal formula, is commonly used for cardiovascular diseases, but its molecular mechanisms remain elusive. Emerging evidence indicates that competing endogenous RNA (ceRNA) networks play critical roles in atherosclerosis pathogenesis. In this study, we constructed an EndMT-associated ceRNA network during atherosclerosis progression by integrating gene expression profiles from the Gene Expression Omnibus (GEO) database and weighted gene co-expression network analysis. Functional enrichment analysis revealed this EndMT-related ceRNA network is predominantly involved in inflammatory responses. ROC curve analysis showed the identified hub genes can effectively distinguish between normal vasculature and atherosclerotic lesions. Furthermore, Kaplan-Meier analysis demonstrated that high expression of IL1B significantly predicts ischemic events in atherosclerosis. Molecular docking revealed most DSY bioactive components can bind key EndMT-related lncRNAs, including AC003092.1, MIR181A1HG, MIR155HG, WEE2-AS1, and MIR137HG, suggesting DSY may mitigate EndMT in atherosclerosis by modulating the ceRNA network.

## 1 Introduction

Atherosclerosis is a chronic inflammatory disease characterized by plaque formation, hardening, and thickening of the vessel wall. As a predominant risk factor for cardiovascular diseases that are now the leading cause of mortality and morbidity worldwide, atherosclerosis encompasses multiple pathological processes, yet is initiated by endothelial dysfunction (1, 2). Under physiological conditions, endothelial cells lining the interior surface of blood vessels regulate the passage of ions and fluid. However, under the influence of pro-inflammatory cytokines, endothelial cells progressively acquire mesenchymal traits, a process termed endothelial-to-mesenchymal transition (EndMT). During EndMT, endothelial cells lose expression of junctional proteins including VE-cadherin, platelet endothelial cell adhesion molecule-1(PECAM-1) and claudin-5, alongside endothelial markers such as CD31, Tie-2 and endothelial nitric oxide synthase (eNOS). Concurrently, they gain mesenchymal markers including ferroptosis suppressor protein 1(FSP1), vimentin and alpha-smooth muscle actin(α-SMA), while transforming from apical-basal polarized cobblestone-shaped cells into spindle-shaped, motile and invasive ones. EndMT gives rise to extracellular matrix deposition of collagen I, collagen III, laminin and fibronectin, thereby promoting vascular fibrosis and plaque formation.

With the aid of genetic lineage tracing tools, EndMT has been demonstrated as a major driving force underlying the initiation and progression of atherosclerosis. For instance, Evrard et al. revealed an abundance of EndMT-derived fibroblast-like cells within atherosclerotic lesions, indicative of EndMT's contribution to atherosclerosis (3). Additionally, EndMT has been implicated in plaque calcification. Matrix metalloproteinases (MMPs) are associated with unstable atherosclerotic lesions (4, 5), and EndMT-derived fibroblast-like cells exhibit higher expression of MMP1, MMP9 and MMP10 compared to normal fibroblasts (6). A greater proportion of EndMT-derived fibroblast-like cells are also present in ruptured vs. non-ruptured plaques (3), linking the extent of EndMT to an unstable plaque phenotype. Together, these findings underscore the significance of EndMT in atherosclerosis pathogenesis and support the potential therapeutic value of targeting this process.

Dan-Shen-Yin (DSY) is a well-known traditional Chinese formula composed of Salvia miltiorrhiza, sandalwood, and Fructus Amomi. It was originally documented in Shi Fang Ge Kuo, has been widely used to treat cardiovascular diseases for centuries. Studies have shown that DSY has protective effects against inflammation and reduces oxidative stress in rat models of diabetic atherosclerosis (7), acute ischemic myocardial injury (8), and hypoxia-induced pulmonary hypertension (9). Active ingredients of Salvia miltiorrhiza, including tanshinone IIA (10), and salvianolic acid A (11), can effectively treat atherosclerosis. Extracts from sandalwood, and Fructus Amomi were also demonstrated to inhibit inflammation (12, 13), indicating their underline role in the treatment of atherosclerosis. We previously demonstrated that DSY could inhibit inflammatory cytokines induced EndMT, suggesting the DSY may treat atherosclerosis through targeting EndMT, but the precise active components and their mechanisms need to be further explored (14). Further research on DSY is warranted to understand its therapeutic effects on endothelial function and atherosclerosis and a deeper understanding of DSY may uncover novel approaches for preventing and treating atherosclerotic cardiovascular diseases.

The competitive endogenous RNA (ceRNA) regulatory network has recently emerged as a potential novel mechanism underlying improved atherosclerosis therapy. The ceRNA hypothesis was first proposed by Salmena et al. as a unique mode of interaction between non-coding RNAs (ncRNAs) and messenger RNAs (mRNAs) (15). MicroRNA (miRNA) response elements (MREs), which act as ceRNAs and play critical roles in various pathological processes, are present within long non-coding RNAs (lncRNAs), pseudogene transcripts, circular RNAs (circRNAs), viral RNAs, and protein-coding transcripts (15, 16). miRNAs are a diverse group of small non-coding RNA molecules that naturally regulate the expression of target genes by inhibiting translation (17). lncRNAs have been demonstrated to play a role in various cellular processes such as protein scaffolding (18, 19). Recent evidence suggests that lncRNAs can also act as sponges for miRNAs, participating in a wide array of biological processes (17). With advances in molecular detection technologies, increasing evidence demonstrates that most lncRNAs are implicated in atherosclerosis pathogenesis through the lncRNA-miRNA-mRNA (20) axes. However, the precise ceRNA network implicated in EndMT during atherosclerosis progression remains unknown.

Weighted gene co-expression network analysis (WGCNA) is a systems biology approach that identifies clusters of highly correlated genes associated with clinical phenotypes, enabling discovery of potential biomarkers and therapeutic targets for diseases (21). While previous studies have utilized WGCNA to explore differential gene expression in atherosclerosis (22, 23), the key genes driving EndMT and atherosclerosis progression remain unclear. To address these gaps, we harnessed WGCNA to identify EndMT-related genes in atherosclerosis, and further built ceRNA networks to unravel the intrinsic molecular mechanisms of EndMT-mediated atherosclerotic pathogenesis. This integrative approach provides a foundation to guide biologically-targeted EndMT therapies for combating atherosclerosis.

## 2 Methods

### 2.1 Data acquisition and processing

The gene expression datasets-GSE100927 and GSE118446—were obtained from the National Center for Biotechnology Information (NCBI) Gene Expression Omnibus (GEO) database (http://www.ncbi.nlm.nih.gov/geo). The GSE100927 dataset contains 104 human peripheral artery samples from carotid, femoral and infra-popliteal territories in atherosclerotic and control tissues (24). The platform used was GPL17077. Female samples were excluded due to the limited number and potential gender differences in atherosclerosis severity, leaving 85 samples for analysis. GSE118446 contains data on human umbilical vein endothelial cells (HUVECs) treated with transforming growth factor-β2 (TGF-β2), interleukin-1β (IL-1β), or both.

### 2.2 Construction of weighted gene co-expression network

Using the WGCNA package in R software, we constructed a gene co-expression network from GSE100927. As a first step, we filtered for the top 50% most variable mRNAs to focus our analysis. Next, we screened power parameters from 1 to 20 with the pickSoftThreshold function and opted for a soft threshold of 10, which met the independence criterion of 0.85 while having the lowest power value. We employed the Dynamic Tree Cut method to delineate modules, setting deepSplit at 2 and minModuleSize at 30 to avoid generating too many tiny modules. Modules with similarity exceeding 0.75 were consolidated, and we used a height cut-off of 0.25. Therefore, mRNAs clustered within the same module are deemed to be highly co-expressed. We conducted gene ontology biological process enrichment analysis for each module and summarized the most significantly enriched terms in Supplementary Table S1.

### 2.3 Enrichment analysis

To further visualize the activities of genes in the key module, Gene Ontology (GO) and Kyoto Encyclopedia of Genes and Genomes (KEGG) enrichment analyses were performed on hub genes in the cluster Profiler. The cutoff threshold was set at a *P*-value of less than 0.05. ClueGo classified the signal pathways discovered by enrichment analysis into groups based on functional connection; the same group was colored the same color, and the labels of each group of the most essential terms were color-coded.

### 2.4 Evaluating the diagnostic performance of hub genes using ROC curve analysis

The receiver operating characteristic (ROC) curve is commonly used to evaluate the diagnostic accuracy of prediction models (25). The ROC curve plots sensitivity on the vertical axis and 1-specificity on the horizontal axis. Unlike metrics that can be affected by the distribution of positive and negative samples, the ROC curve provides a robust measure of model performance. ROC curves were generated for the expression levels of hub genes using the pROC package. The area under the ROC curve (AUC) and 95% confidence intervals were calculated to quantify the diagnostic power of the hub genes in each gene set. An AUC of 1 represents perfect diagnostic ability, though this is generally theoretical. AUCs between 0.5 and 1 indicate the model is better than random guessing and has predictive value. An AUC of 0.5 means the model has no diagnostic utility. If the 95% confidence interval contains 0.5, the ROC curve lacks statistical significance.

### 2.5 Construction of competing endogenous RNA

RNA-RNA and RNA-protein interaction data were obtained from the RNA Interactome Database (RNAInter) (26) and starBase (27). The top 10 genes in the MEturquoise module were individually entered into the RNAInter website to retrieve competing endogenous RNA (ceRNA) network relationships. Only interactions with a confidence score greater than 0.3 were included. Visualization of the interacting genes with confidence scores exceeding 0.5 was then performed using Cytoscape.

### 2.6 Validation

We validated key genes, lncRNAs, and miRNAs using several datasets from the GEO database. The details about the databases can be found in Supplementary Tables S2, S3.

### 2.7 Molecular docking

The FASTA sequences of lncRNAs were obtained from the NCBI database. The minimum free energy secondary structures of lncRNAs were predicted using the online tool RNAfold, and based on these secondary structures, the 3D structures of lncRNAs were constructed using the web server 3dRNA. During 3D structure prediction, the coarse-grained energy function was optimized with or without residue interactions or distance constraints, and the ff14SB force field was used to minimize the predicted structure energies. Default values were chosen for other parameters. Compared to existing evaluation methods, 3dRNAScore more effectively ranks RNA models towards their near-native states by combining distance-dependent and 2D structure-dependent energies. Lower 3dRNAScore represents better model quality. Thus, the conformation with the lowest score was chosen for analysis. Molecular docking was performed using autodock vina (28). Since the spatial structures of lncRNAs exceeded the maximum volume of the docking box in autodock vina, the sailvina script was used to calculate potential docking sites on the lncRNAs based on their spatial structures before docking (29). Docking boxes of 30*30*30 were then constructed centered on the potential docking sites for docking using autodock vina. Autodock vina uses molecular virtual docking binding affinity to describe docking results. Lower affinity indicates better docking results and higher likelihood of binding. Affinity < −4 kcal/mol is generally considered to have binding forces, while < −7 kcal/mol belongs to strong binding forces. The binding modes between active components and amino acid residues were calculated using autodock vina and Pymol. The binding modes between active components and lncRNAs were computed and illustrated using Discovery Studio.

## 3 Results

### 3.1 Construction of co-expression modules and identification of key module in atherosclerosis

**Figure 1:**
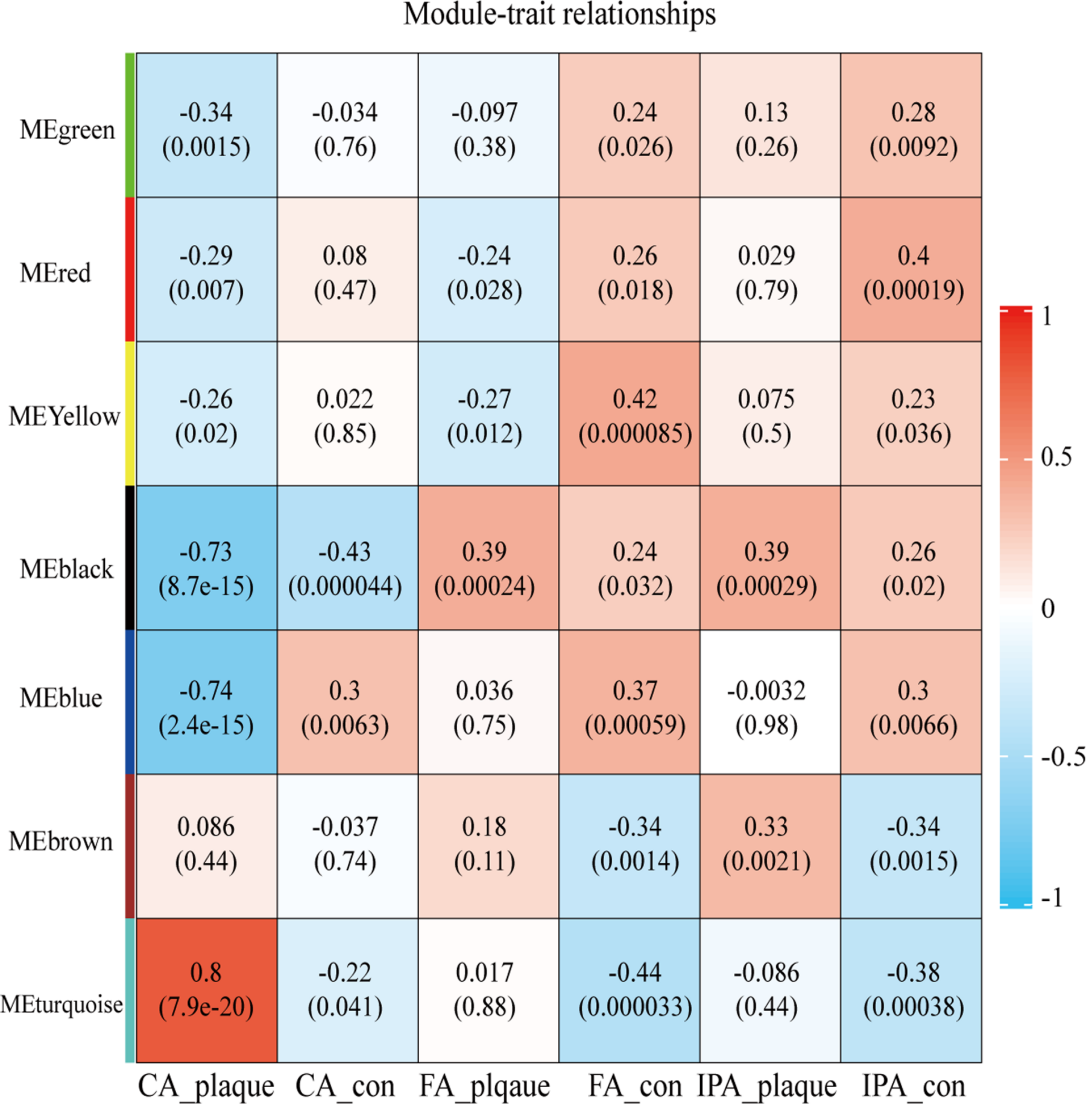
Heatmap depicting correlations between module eigengenes and atherosclerotic plaque. Each row corresponds to one module eigengene, and each column to a trait. The cells present the correlation coefficients (top) and *P*-values (bottom), color-coded based on the legend. *P*-values < 0.05 indicate statistical significance.

After clustering all samples, two outliers in GSE100927 were identified (samples GSM2696641 and GSM6296632) as shown in Supplementary Figure S1. To generate an approximately scale-free topology, an R2 cutoff of 0.83 was employed. Subsequently, dynamic tree cutting was utilized to produce co-expression modules, resulting in 7 modules spanning 103–4,869 genes within the co-expression network (Supplementary Figures S2A,B). The interactions and connectedness between the eigengenes of the various gene co-expression modules were depicted in Supplementary Figure S2C. We then determined and charted the association of each module with their respective clinical characteristics. The most significantly enriched biological process terms associated with each module were outlined in Supplementary Table S1. Figure 1 allowed us to infer that the turquoise module demonstrated the strongest positive correlation (module-trait weighted correlation = 0.8, *P* = 7.9E-20), blue and black module indicated the strong negative correlation (module-trait weighted correlation = −0.74, *P* = 2.41E-15; module-trait weighted correlation = −0.73, *P* = 8.7E-15 independently). Turquoise, black and blue module were pinpointed as the pivotal module for plaque progression during atherosclerosis.

### 3.2 Identification of key genes involved in EndMT and atherosclerosis

**Figure 2:**
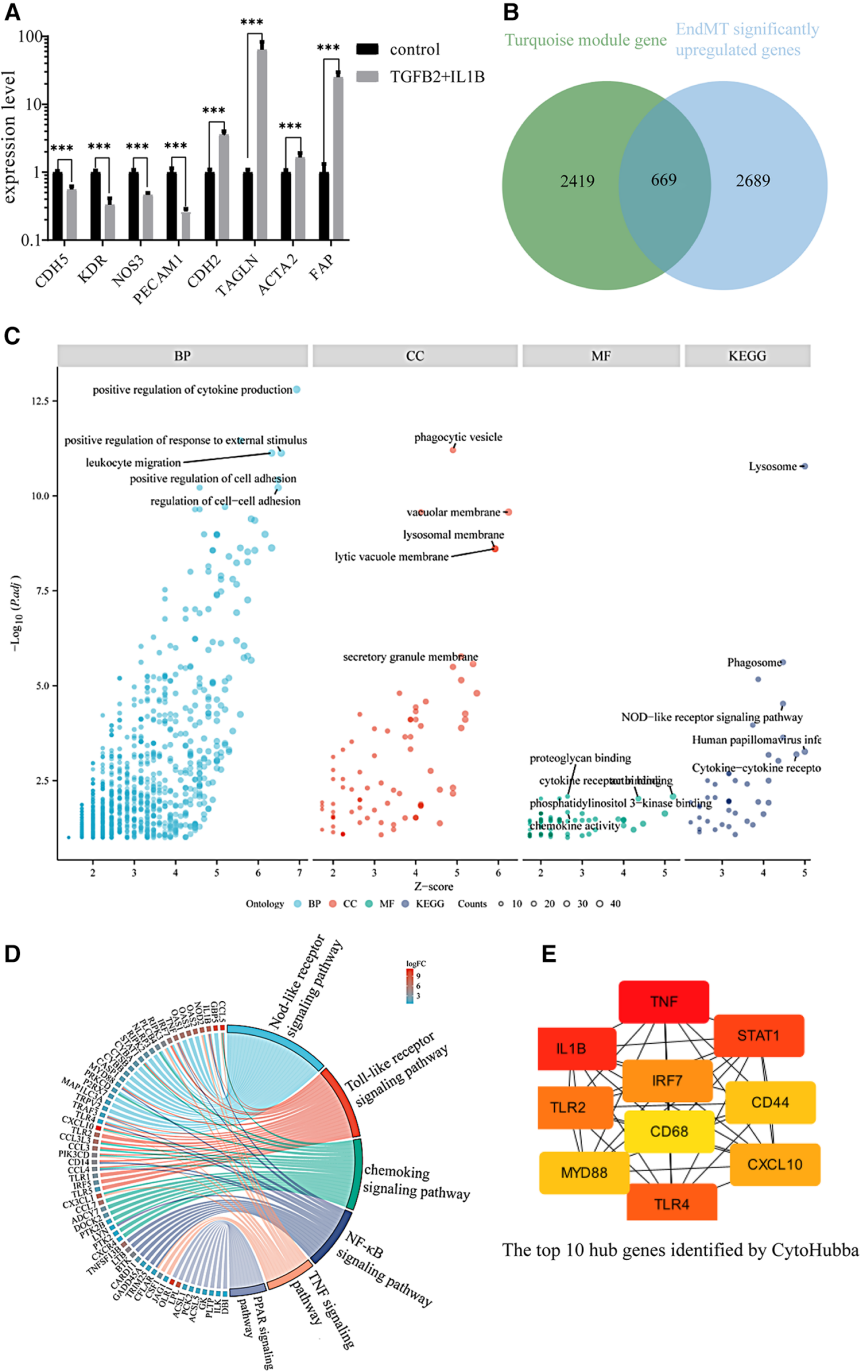
Identification of the key genes involved in endMT and atherosclerosis. (**A**) The expression of EndMT markers in the GSE118446 dataset. (**B**) Illustrates the intersection between the turquoise module and up-regulated genes in the GSE118446 dataset. (**C**) The enrichment analysis of the intersected genes. BP: Biological processes, CC: cellular components, MF: molecular functions. Higher adjusted *p*-values indicate greater enrichment of the terms. Higher *z*-scores indicate greater up-regulation of the terms. The size of the dots represents the number of genes in each term. (**D**) the pathway enrichment analysis of the intersected genes. The left side presents the genes, with each color block depicting the corresponding log2FC value. The right side exhibits the enriched pathway terms, where the size of the color blocks represents the number of genes in each pathway. (**E**) the top 10 hub genes identified by CytoHubba.

To further analyze if key genes are involved in regulating EndMT in atherosclerosis, we downloaded the GSE118446 dataset for further analysis. The combination treatment of TGF-β and IL-1β was potent inducer for EndMT (30), markedly changed the expression of EndMT markers in the dataset of GSE118446 as shown in Figure 2A, indicating this dataset underwent EndMT. The intersection of downregulated genes in GSE118446 with genes in the black and blue modules resulted in 31 and 135 genes, respectively. After removing genes that did not meet the adjusted *p*-value <0.05 and log2FC >0.58 thresholds, only 15 and 44 genes remained in the intersecting gene sets from the black and blue modules, respectively. This resulted in protein-protein interaction (PPI) networks containing only 6 nodes and 6 edges for black module, 13 nodes and 10 edges for blue module (Supplementary Figure S3). Due to the small number of interactions, these networks were not suitable for further analysis. Therefore, only the data from the turquoise module will be used for subsequent analysis. The enrichment analysis of turquoise module is shown in Supplementary Figure S4.

The intersection of upregulated genes in GSE118446 with genes in turquoise module resulted in 2,357 genes. Among these, there are 669 genes that satisfy adjust-*P* < 0.05 and log2FC > 0.58 (Figure 2B). The shared genes of GSE118446 and turquoise module were input into the BisoGenet plugin in Cytoscape to create a PPI network containing 666 nodes and 2,903 edges. The candidate targets of the PPI network were then explored by GO and KEGG enrichment analysis. The GO category results suggested that the predicated targets were highly involved in multiple biological processes including positive regulation of cytokine production, positive regulation of responses to external stimuli, leukocyte migration, and cell adhesion; and phagocytic vesicle, vacuolar membrane, lysosome membrane, lytic vacuole membrane, and secretory granule membrane in the cellular component (CC) category; and proteoglycan binding, cytokine receptor binding, phosphoinositide 3 kinase binding, chemokine activity in the molecular function (MF) category; and lysosome, phagosome, NOD-like receptor signaling pathway, human papillomavirus infection, cytokine-cytokine receptor in the KEGG category (Figure 2C). Additionally, pathway enrichment analysis of the intersecting genes revealed involvement in the NOD-like receptor signaling pathway, Toll-like receptor signaling pathway, chemokine signaling pathway, nuclear factor kappa-B (NF-kB) signaling pathway, tumor necrosis factor (TNF) signaling pathway, and peroxisome proliferators-activated receptor (PPAR) signaling pathway (Figure 2D). Finally, we found that the top 10 connected hub genes were TNF, IL1B, signal transducerand activator of transcription 1 (STAT1), toll-like receptor 4 (TLR4), toll-like receptor 2 (TLR2), interferon regulatory factor (IRF7), interferon regulatory factor (CXCL10), myeloid differentiation factor88 (MYD88), CD44, CD68 (Figure 2E), indicating these 10 genes maybe the potential regulators of EndMT during atherosclerosis.

**Figure 3:**
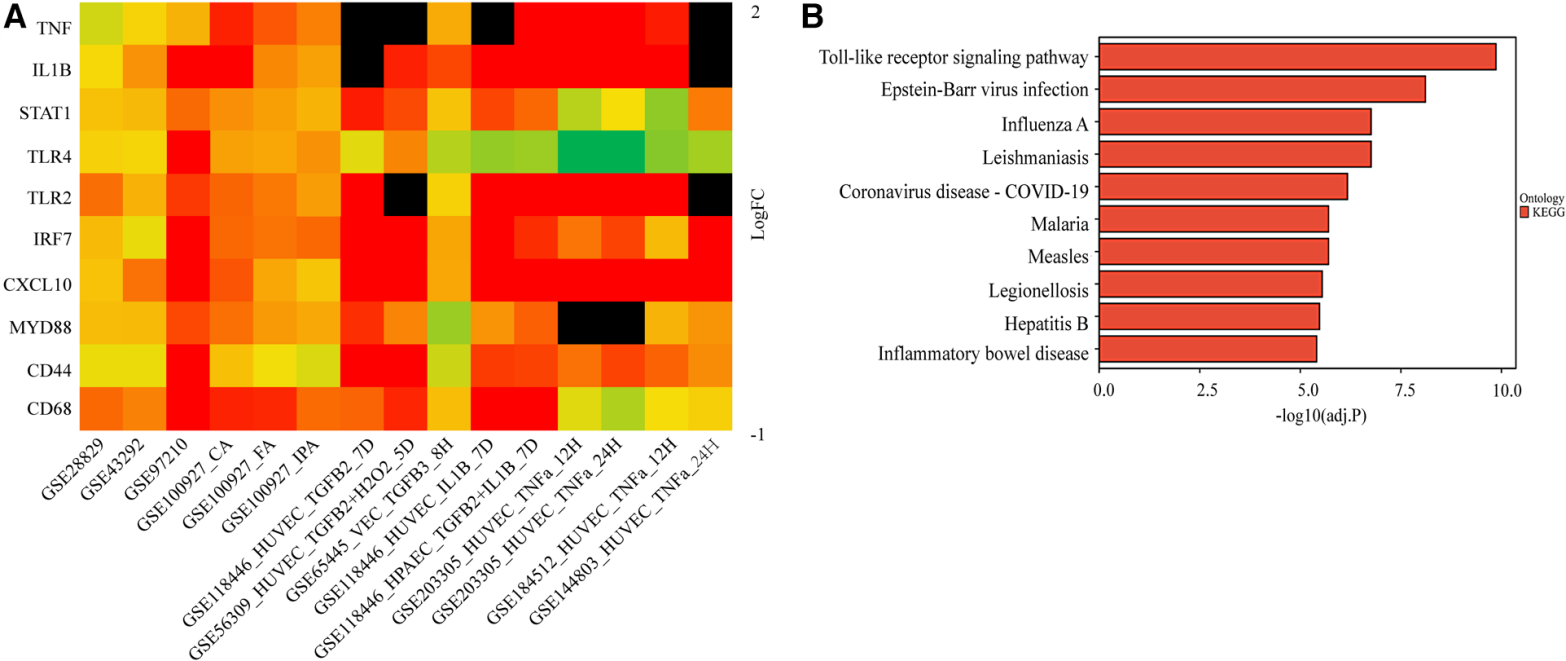
Validation of the hub genes involved in endMT and atherosclerosis. (**A**) The log2FC heatmaps of the hub gene expression across the various validation datasets. Red indicates up-regulation, green indicates down-regulation, and darker colors represent higher expression levels. Black indicates the gene is missing in the validation set. (**B**) The KEGG enrichment of the hub genes.

We further validated their expression across multiple datasets from GEO. The occurrence of EndMT in the EndMT validation datasets was confirmed by analyzing the expression level of EndMT marker genes as shown in Supplementary Figure S5. Figure 3A and Supplementary Figure S5 showed that TLR4 expression was mostly downregulated in the dataset of endothelial cells treated with inflammatory cytokines, suggesting inconsistent TLR4 expression. Therefore, TLR4 was excluded from further analysis. KEGG enrichment analysis was performed on the identified hub genes, and the top-ranking pathway was the Toll-like receptor signaling pathway, as shown in Figure 3B. The positions of the hub genes in the Toll-like receptor pathway are depicted in Supplementary Figure S6.

**Figure 4:**
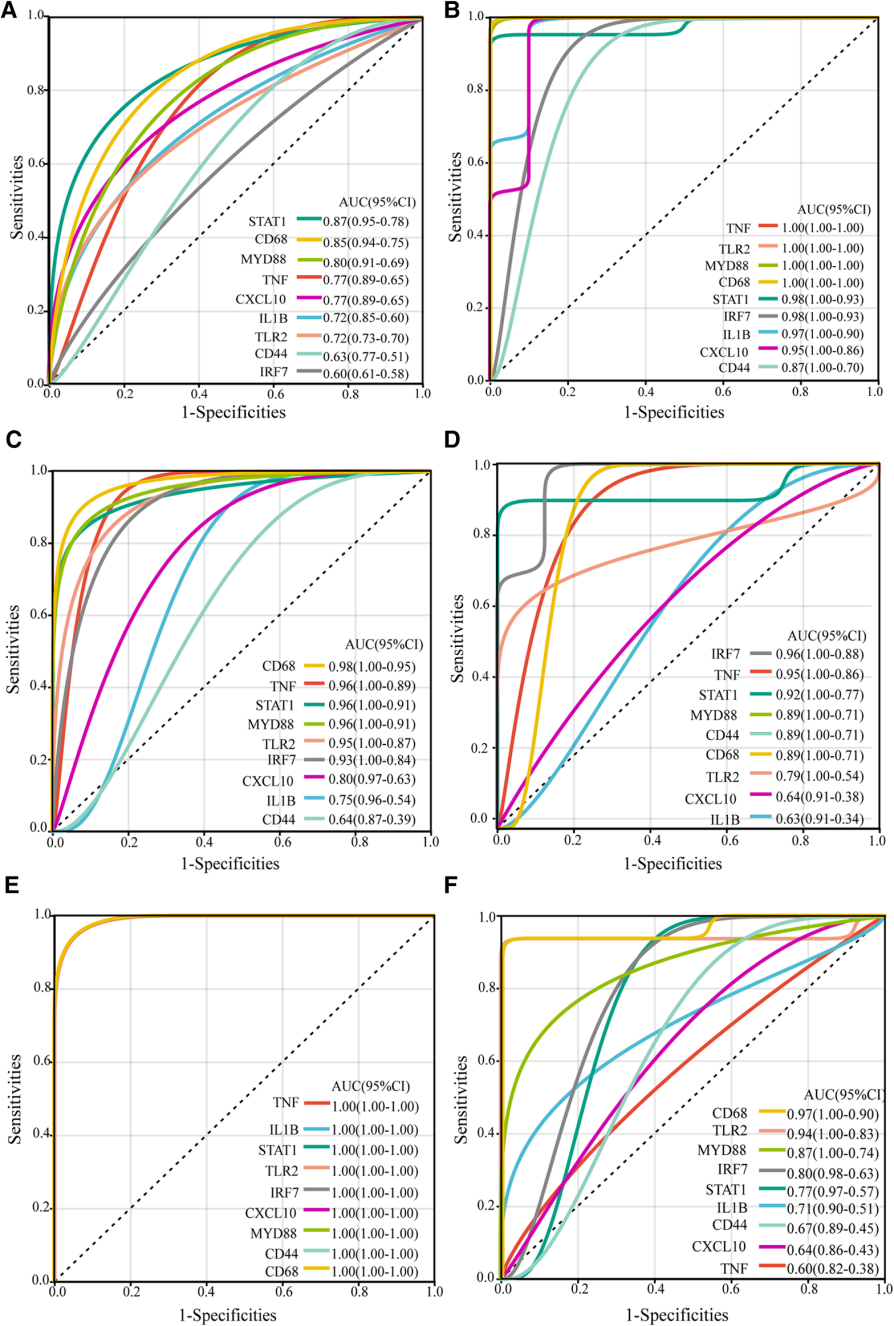
ROC curves of the hub genes in validation datasets. The ROC curves for the hub genes in dataset GSE43292 (**A**), in carotid plaque samples from dataset GSE100927 (**B**), in femoral plaque samples from GSE100927 (**C**), in popliteal plaque samples from GSE100927 (**D**), in dataset GSE97210 (**E**), and in dataset GSE28829 (**F**) The figure shows ROC curves, with 1-specificity (the ability to correctly identify negative samples) on the x-axis and sensitivity (the ability to correctly identify positive samples) on the y-axis. The colored lines represent ROC curves for each hub gene. The lower left corner of each plot provides a color legend and the sorted area under the curve (AUC) values, along with confidence intervals for the AUC.

To further evaluate the diagnostic potential of the identified hub genes in atherosclerosis, receiver operating characteristic (ROC) curve analysis was performed. As depicted in the Figure 4, the hub genes demonstrated AUC values greater than 0.6 in the GSE43292, GSE100927, and GSE97210 validation cohorts, suggesting superior discrimination of atherosclerotic plaques from normal tissue compared to random chance. Moreover, analysis of the GSE28829 dataset revealed CD68, TLR2, MYD88, IRF7, STAT1, and IL1B had statistically significant AUCs capable of excellently distinguishing advanced atherosclerotic plaques from early lesions (AUC > 0.7). In summary, ROC curve analysis verified the hub genes have significant diagnostic value in detecting atherosclerotic plaques and differentiating advanced lesions from early plaque development.

**Figure 5:**
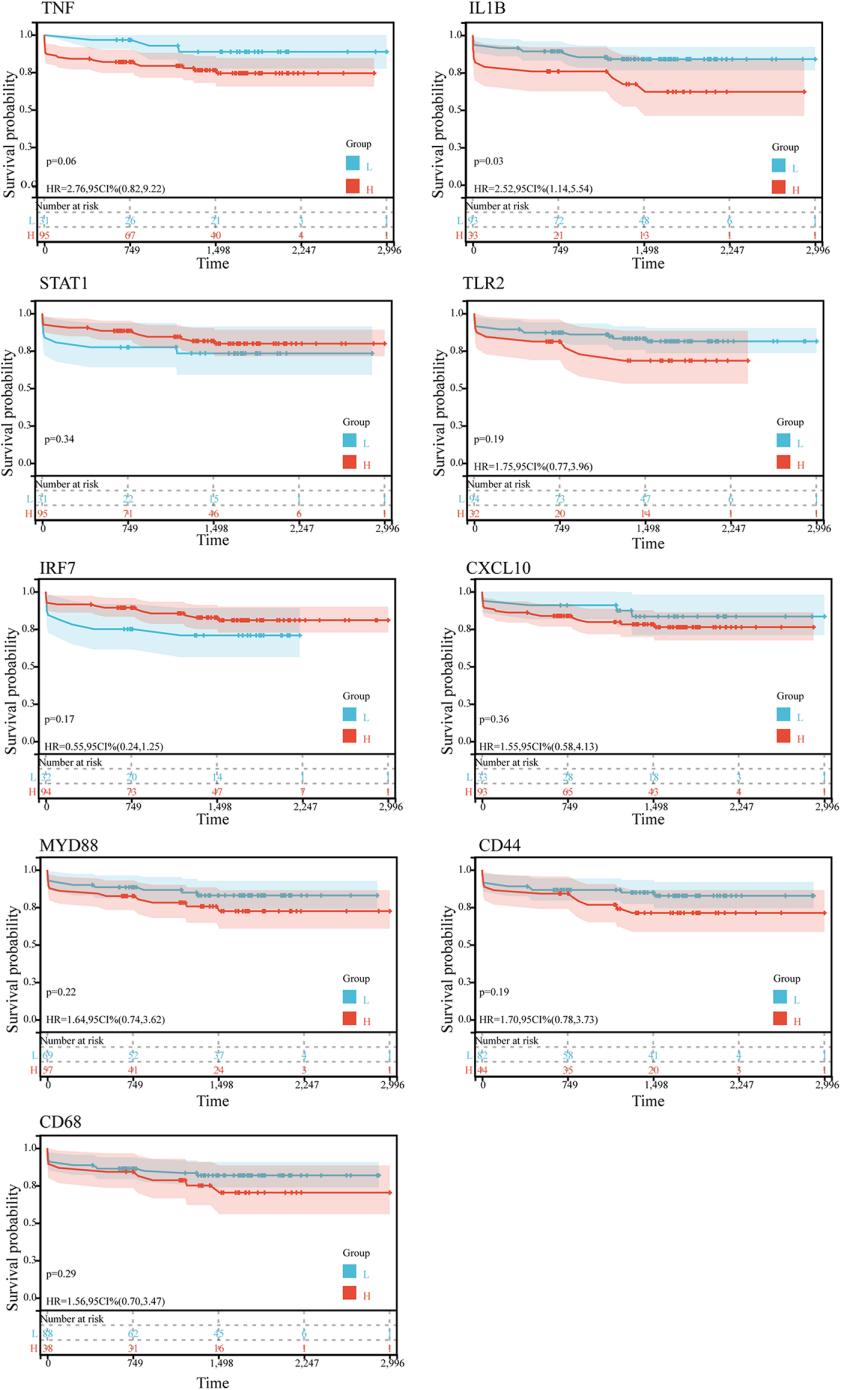
Kaplan-Meier survival curves of 9 hub genes in ischemic event in patients suffer from atherosclerosis in GSE21545. The *P*-value was calculated by the log-rank test.

Kaplan-Meier survival analysis was conducted to evaluate the association between hub gene expression and ischemic event risk in atherosclerosis. As shown in the Figure 5, IL1B was the only hub gene demonstrating a significant impact, with the high IL1B expression group showing a markedly increased probability of ischemic events compared to the low expression group (*P* < 0.05). The risk of ischemic events was 2.52 times higher in the high vs. low IL1B groups. In contrast, the other hub genes did not significantly influence ischemic event occurrence. The lack of association for the remaining hub genes may be attributed to alternative mechanisms governing atherosclerotic ischemic events.

### 3.3 CeRNA network regulating EndMT in as plaque development

TNF, IL1B, STAT1, TLR2, IRF7, CXCL10, MYD88, CD44, and CD68 were identified as hub genes capable of co-regulating atherosclerosis and EndMT. A total of 1,453 high-confidence interactions (score ≥ 0.3) were extracted from 50,499 total relationships in the RNAInter database. By integrating these data with the Starbase database, an mRNA-miRNA network was constructed in Cytoscape and filtered to 60 miRNAs with degree ≥3. Mining the RNAInter and Starbase database further yielded 174 circRNA-miRNA pairs and 5,483 lncRNA-miRNA relationships. The resulting mRNA-miRNA-lncRNA/circRNA network contained 9 mRNAs, 60 miRNAs, 1,532 lncRNAs, and 26 circRNAs.

**Figure 6:**
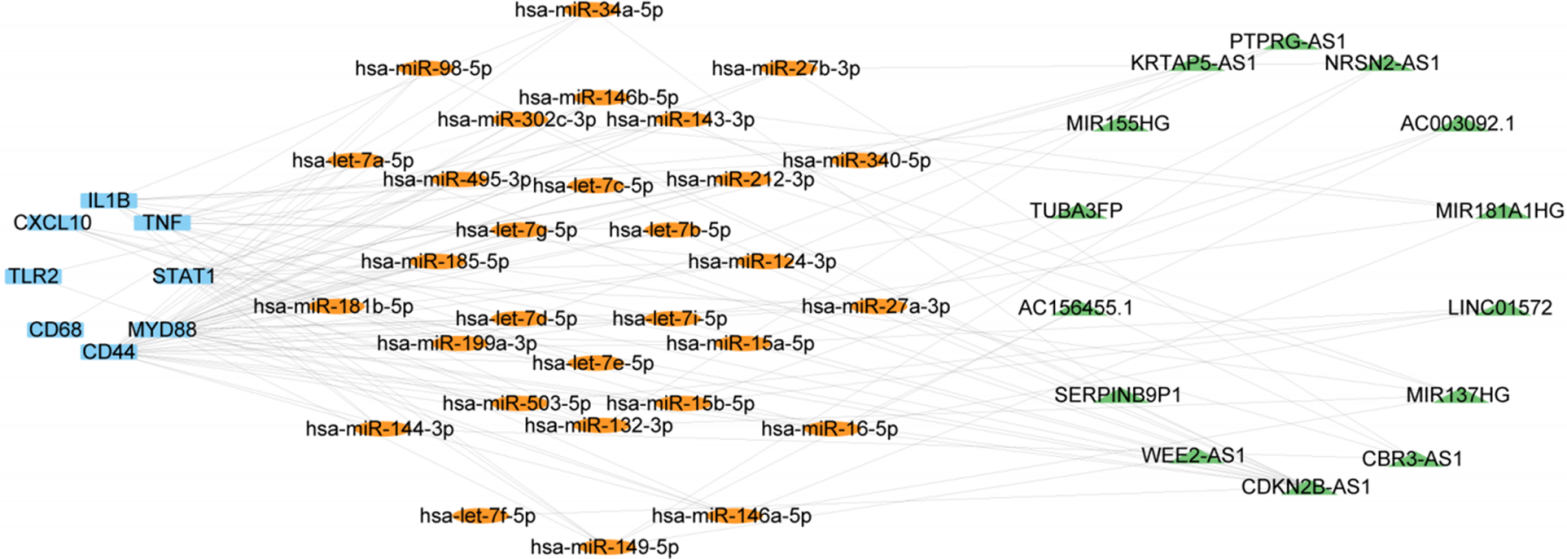
ceRNA network consisting of 14 lncRNAs, 30 miRNAs, and 9 mRNAs. Nodes are color-coded—blue for mRNAs, orange for miRNAs, and green for lncRNAs.

We next validated the expression level of miRNA, LncRNA and cirRNA using the atherosclerosis and EndMT validation datasets (Supplementary Tables S2, S3). The occurrence of EndMT in the EndMT validation datasets was validated by analyzing the expression levels of EndMT marker genes (Supplementary Figure S7). From Supplementary Figure S8A, 33 of the 60 miRNAs obtained from the RNAInter database showed a mostly downregulated trend, consistent with expectations. These 33 miRNAs could regulate all 9 hub genes. lncRNAs that were upregulated in all 5 validation sets and had at least one dataset with LogFC > 1 were selected. 14 lncRNAs met the criteria, and their expression heatmaps were presented in Supplementary Figure S8B. The circRNAs obtained from the RNAInter database had many missing values in the validation sets. Also, there were few circRNA datasets related to atherosclerosis in the GEO database, and the data quality was poor. Thus, the analysis results may have large errors. Therefore, we focused on constructing an mRNA-miRNA-lncRNA network in the subsequent ceRNA network analysis. The validated mRNAs, miRNAs, and lncRNAs were used to construct an mRNA1-miRNA1-lncRNA1 network in Cytoscape (Figure 6). This network contained 9 mRNAs, 30 miRNA1s and 14 lncRNA1s. The miRNAs regulated the hub genes except for IRF7.

### 3.4 Construction of ceRNA network of DSY in inhibiting EndMT in the treatment of athersclerosis

**Figure 7:**
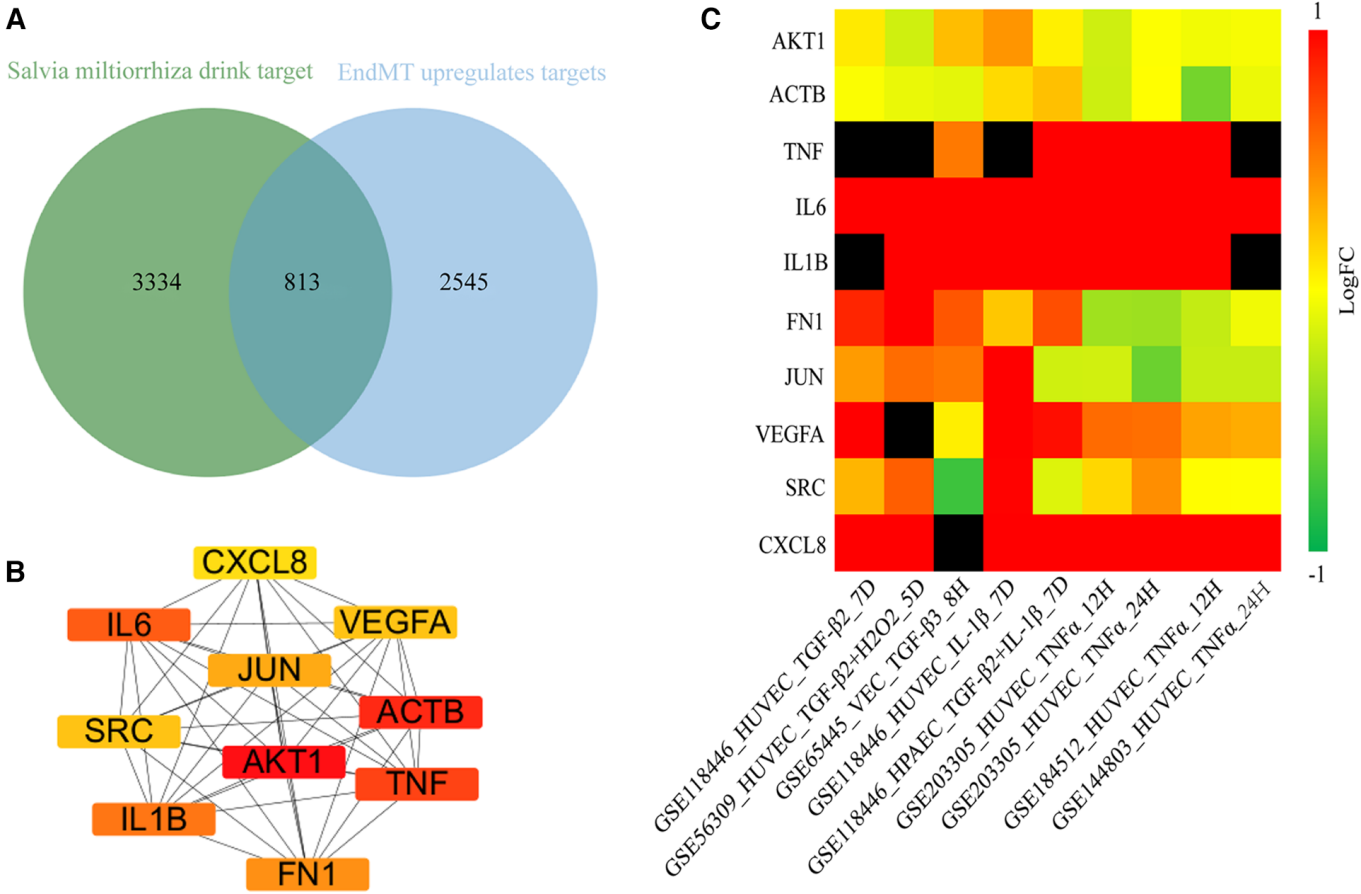
Identification of hub genes regulated by DSY in endMT. (**A**) Venn diagram showing the intersection between genes targeted by DSY and significantly upregulated EndMT-associated genes in the GSE11846 dataset. (**B**) Top 10 hub2 genes ranked by degree centrality, with redder colors indicating higher degree values. (**C**) Expression levels of the identified hub2 genes across EndMT validation datasets.

Next, we aimed to elucidate the ceRNA mechanism through which DSY regulates EndMT to treat atherosclerosis. The analysis rationale is illustrated in Supplementary Figure S9. As demonstrated previously, a total of 227 ingredients and 4,147 gene targets were screened in Dan Shen Yin (DSY) (14). As illustrated above, 3,358 upregulated genes were identified in the EndMT ongoing dataset (GSE118446), among which 813 genes overlapped with DSY targets. PPI network analysis further screened the top 10 hub genes, namely AKT1, actin beta gene (ACTB), TNF, IL6, IL1B, fibronectin1 (FN1), JUN, vascular endothelial growth factor A (VEGFA), SRC, and CXCL8 (Figure 7B). Validation in multiple EndMT datasets showed that AKT1 and ACTB were not markedly upregulated among the EndMT validation datasets, whereas TNF, IL6, IL1B, VEGFA, and CXCL8 were significantly upregulated across validation sets. In contrast, JUN, FN1, and SRC were upregulated specifically in the TGF*-*β2 and IL-1β induced EndMT validation set (Figure 7C). Consequently, AKT1 and ACTB were removed from the analysis, while the remaining hub genes were retained as hub2 for subsequent analysis.

Next, 74 and 231 mRNA2-miRNA2 pairs were obtained from RNAInter and Starbase individually, amounting to 305 miRNA2 in total. Among these 305 miRNA2, 24 overlapped with miRNA1, and these overlapping miRNAs were defined as miRNA3. It was found that miRNA3 could regulate all Hub2 genes. A network comprising DSY active components, mRNA2 and miRNA3 was then constructed as illustrated in Supplementary Figure S10. In this network, miRNA3 exhibited correlations with DSY active components, suggesting that miRNA3 may be the mechanism underlying DSY active components' regulation of EndMT occurrence/development and atherosclerosis progression.

**Figure 8:**
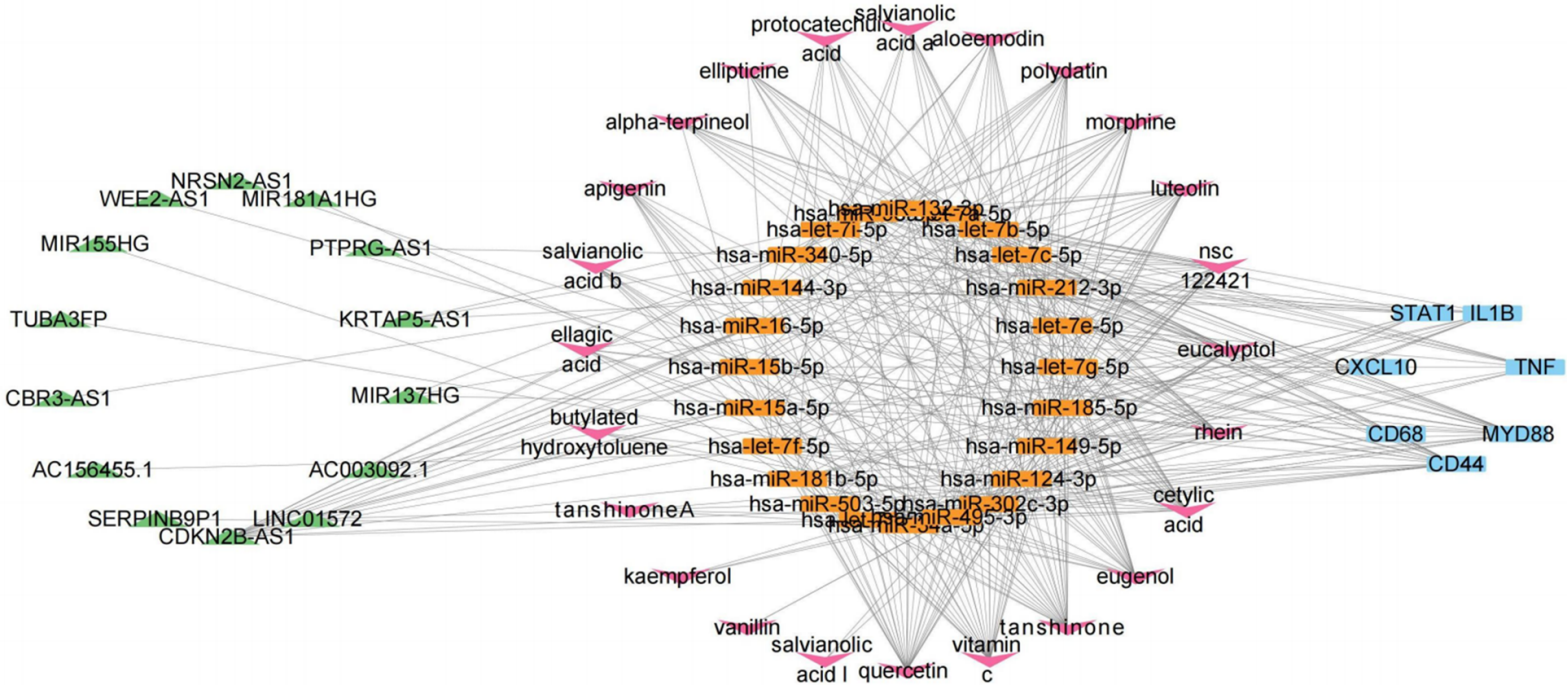
CeRNA network of lncRNA1-miRNA3-mRNA1-active components of DSY.

To investigate the potential lncRNA targets of DSY active components, we integrated miRNA3 back into the mRNA1-miRNA1-lncRNA1 network and identified lncRNAs associated with miRNA3. Results revealed that miRNA3 had direct regulatory effects on all lncRNA1. Consequently, these lncRNAs also exhibited indirect correlations with DSY active components. A network comprising lncRNA1, miRNA3 and DSY active components was constructed as depicted in Figure 8, implying DSY active components could suppress EndMT and thereby treat atherosclerosis through interactions within this network. This network contained 24 miRNA3, 14 lncRNA1 and 36 DSY active components. Among them, 24 DSY components had degree values ≥2, denoting associations of these 24 active components with 2 or more miRNA3. These 24 bioactive components of DSY were selected for subsequent analyses.

To further explore whether the active components of DSY can directly act on lncRNA1, molecular docking was performed between the 24 active components of DSY and lncRNA1. To select lncRNAs with research significance, lncRNA expression levels in the validation sets were considered, and the top 50% lncRNAs were screened. In the ceRNA network, KRTAP5-AS1, AC003092.1, MIR181A1HG, MIR155HG, WEE2-AS1, and MIR137HG were ranked at the top for their significantly upregulated expression (adjust-*P* < 0.05) across atherosclerosis and EndMT validation sets, and their expression levels are shown in Supplementary Figure S11. The 3D structures of the lncRNAs were successfully established. Based on the spatial structures of lncRNAs, AC003092.1 had 1,232 potential docking sites, KRTAP5-AS1 had 3,328, MIR137HG had 5,376, MIR155HG had 3,094, MIR181HG had 880, and WEE2-AS1 had 2,772 potential docking sites. The docking results are shown in Supplementary Table S4. It can be seen that most active components had strong binding affinity (affinity ≤ −7 kcal/mol) to lncRNAs, suggesting the active components may act on lncRNAs and thus exert biological effects. Among the effective components with binding affinity ≤ −7 kcal/mol, active components from Salvia miltiorrhiza accounted for the majority, but some compounds from sandalwood, and Fructus Amomi also showed good binding affinity, such as astragalin and tannic acid. Quercetin, luteolin and apigenin are shared components across the three constituents of DSY, and they also exhibited strong binding to lncRNAs. The binding modes between lncRNAs and the top three active components with best docking binding affinity were depicted in Supplementary Figure S12. Active compounds from DSY that could strongly bind to lncRNA3 and form hydrogen bonds were selected to construct a compound-lncRNA3-miRNA1-mRNA gene network (Supplementary Figure S13). These compounds may influence the sponge activity of lncRNA3 by binding to it, and thereby regulate the key EndMT-atherosclerosis gene Hub1 through miRNA-mediated regulation.

The binding affinities of active components with hub1 gene were also determined, and the heat map of binding energies is shown in Supplementary Figure S14. The binding energies of most active compounds from Salvia miltiorrhiza with hub genes were lower than −7 kcal/mol, indicating Salvia miltiorrhiza may be the major active herb in DSY. Gallic acid, oleuropein, eugenol, α-tocopherol, vitamin C, palmitic acid, p-hydroxytoluene, and paeonol had relatively higher binding energies with most hub genes, suggesting these compounds may not directly bind and act on hub genes. The other compounds may directly target hub genes to inhibit EndMT to treat atherosclerosis.

## 4 Discussion

Atherosclerosis represents the most prevalent cardiovascular disease worldwide, conferring considerable morbidity and mortality. The inner lining of blood vessels, composed of endothelial cells, has recently been demonstrated to undergo transition into mesenchymal cells through a process termed EndMT. During this process, endothelial cells gradually acquire mesenchymal characteristics, known to significantly contribute to the growth of atherosclerotic plaques. EndMT can expedite fibronectin deposition, thus promoting further plaque development (31). Macrophages, which are prevalent in atherosclerotic lesions, can also trigger EndMT. Conversely, EndMT can influence the lipid uptake capability and phenotypes of macrophages, thereby influencing the composition of the atherosclerotic plaque (32). Various stimuli provoke this EndMT process, including growth factors, cytokines, hypoxia, and disturbed flow (33–35). In this study, we constructed a weighted gene co-expression network and identified a competitive endogenous RNA network related to EndMT in AS using WGCNA analysis. This allows us to uncover the intricate molecular events underlying EndMT during AS pathogenesis and try to uncover the material basis of famous Chinese formula DSY in treatment of atherosclerosis. This study focused on the turquoise module from WGCNA. To identify EndMT-related genes impacting atherosclerosis, this study intersected TGF-β2 and IL-1B-induced EndMT-upregulated genes with the turquoise module, analyzing EndMT associated ceRNA network in the pathogenesis of atherosclerosis.

Most of the identified mRNAs in the EndMT related ceRNA network have been reported in atherosclerosis or EndMT related study. TNFα and IL-1β are classic proinflammatory cytokines and common inducers of EndMT, playing inflammatory roles during atherosclerosis development. MyD88 transduces signals in the TLR/MYD88/NF-*κ*B pathway to activate NF-*κ*B and promote inflammation (36). MyD88 and TLR2 participate in LPS induced EndMT (37). MyD88 deficiency prevents early atherosclerosis (38), likely by suppressing inflammation resultant from EndMT. STAT1 facilitates epithelial mesenchymal transition (EMT) (39) and acts as a key effector in the interferon signaling pathway, which activates macrophages via STAT1-dependent mechanism in atherosclerosis (40). CD44 is an adhesion molecule that can recruit activated inflammatory cells adhere to blood vessels, thus promote atherosclerosis (41). The osteopontin/CD44 interaction regulates disturbed flow-induced EndMT, contributing to neointimal hyperplasia in arteriovenous fistulas and representing a potential therapeutic target (42). CD68 is a glycosylated glycoprotein highly expressed in macrophages as a marker. The specific mechanism of CD68 on atherosclerosis remain elusive but CD68-induced changes may relate to macrophage infiltration (43). CXCL10 is a chemokine expressed at all stages of atherosclerosis, participating in VSMC proliferation and intimal hyperplasia (44). Additionally, it can activate the PI3K/AKT pathway via CXCR3, inhibit GSK-3β phosphorylation, leading to Snail upregulation and promoting EMT (45).

In our study, based on bioinformatics and network pharmacology approaches, we further explored the potential ceRNA mechanisms by which DSY may inhibit EndMT during atherosclerosis progression. In the ceRNA network, we identified essential lncRNAs, including KRTAP5-AS1, MIR181A1HG, MIR155HG, WEE2-AS1, MIR137HG, and CDKN2B-AS1. KRTAP5-AS1 can sponge miRNAs like hsa-miR-340-5p to repress their inflammation-related targets IL1B, STAT1, MYD88, CD44. MIR181A1HG regulates miRNAs (e.g., hsa-miR-146a-5p, hsa-miR-146b-5p, hsa-miR-199a-3p, hsa-miR-302c-3p) that suppress TNF, STAT1, MYD88, CD44, TLR2, CXCL10. Although the direct effect of MIR181A1HG on atherosclerosis is unreported, many of its miRNA targets such as miR-146a-5p and miR-199a-2p have been shown to protect endothelial cells and inhibit vascular smooth muscle cell proliferation (46–48). This suggests MIR181A1HG may play a role in atherosclerosis progression through modulating these anti-inflammatory and anti-proliferative miRNAs. MIR155HG plays a pro-inflammatory role by promoting NLRP3, and M2-to-M1 conversion (49, 50). It may also regulate miR-155/SOX10 to mediate TGF-*β*-induced EMT (51). Although unreported in atherosclerosis, MIR155HG has strong potential in the disease onset. WEE2-AS1 sponges hsa-miR-149-5p to repress its targets TNF, IL1B, STAT1, MYD88. WEE2-AS1 is upregulated in arteriosclerosis obliterans and could inhibit endothelial viability (52). miR-149-5p has been shown to regulate endothelial injury, vascular smooth muscle cell proliferation and invasion—processes that are crucial for atherosclerotic development (53, 54). This indicates WEE2-AS1 and miR-149-5p may play important roles in atherosclerosis through their competing endogenous RNA network. In summary, our ceRNA network analysis identified lncRNAs that may promote EndMT and atherosclerosis via inflammation- and EMT/EndMT-related miRNAs.

The DSY bioactive components were confirmed to bind with the identified lncRNAs, and may thereby inhibit EndMT in atherosclerosis by modulating downstream miRNAs and mRNAs. Molecular docking revealed that some DSY active ingredients have poor binding affinity for proteins, but can strongly bind lncRNAs. For example, the ceRNA network showed salvianolic acid B could target IL1β, however salvianolic acid B has low binding affinity for IL1β protein. Instead, it can avidly bind lncRNAs KRTAP5-AS1, MIR137HG and WEE2-AS1, further enabling modulation of hsa-miR-149-5p, hsa-miR-212-3p, hsa-miR-132-3p and hsa-miR-340-3p to regulate IL-1β. This suggests DSY ingredients may act by directly targeting proteins or through ceRNA mechanisms, providing new perspectives on drug mechanism analysis and expanding traditional Chinese medicine applications.

This study successfully identified key genes associated with EndMT-related atherosclerosis, such as CD68, TLR2, MYD88, IRF7, STAT1, and IL1B, all of which demonstrate substantial diagnostic potential for detecting atherosclerotic plaques. Importantly, these genes show promise in distinguishing advanced lesions from early plaque development, suggesting their potential as therapeutic targets for treating atherosclerosis. Furthermore, the construction of a ceRNA network based on these key genes presents an opportunity to explore treatments that target this network, offering the prospect of better understanding and potentially mitigating the impact of atherosclerosis in the future. In addition, our discussion of the renowned formula DSY sheds light on the potential bioactive components that could serve as valuable elements for further study. This opens up new avenues for exploration and potential intervention strategies. However, it is important to acknowledge limitations within this study. First, Branches and curvatures of arteries experience intricate blood flow patterns resulting in low or oscillatory shear stress, fostering a mechanical environment conducive to vascular dysfunction and atherosclerosis (55). However, this paper exclusively addresses the role of inflammatory cytokines in EndMT-associated atherosclerosis, thus not encompassing the entire narrative. In the future, broader scenarios should be contemplated to gain a more comprehensive understanding of the mechanisms governing atherosclerosis. Second, the GEO database lacks datasets comparing normal vascular tissue, early atherosclerotic plaques, and advanced atherosclerotic plaques, which may limit the hub genes identified. The GEO database contains some relevant datasets, including GSE28829 (*n* = 29) comparing early and late-stage plaques, and GSE97210 (*n* = 6) and GSE100927 comparing late-stage plaques to normal vessels. Some researchers have suggested integrating datasets and removing batch effects using Limma package. However, these datasets were generated on different sequencing platforms, leading to platform-dependent differences in expression levels. Merging them may increase errors, so we did not utilize integrated datasets here. Additional, we did not conduct experimental validation or mechanistic studies, so whether the identified associations are causal or consequential remains unclear. Further studies can investigate the interactions in experimental models and verify prognostic values in larger cohorts.

## Data Availability

The original contributions presented in the study are included in the article/Supplementary Materials, further inquiries can be directed to the corresponding author.
