# Supplementary material for: Constructing a competitive endogenous RNA network of EndMT-related atherosclerosis through weighted gene co-expression network analysis
Source: Front Cardiovasc Med. 2024 Jan 10;10:1322252. doi: 10.3389/fcvm.2023.1322252 (PMC10806165; doi:10.3389/fcvm.2023.1322252)
Supplement: Supplementary file 1 [file Datasheet1.docx]

Supplementary Material

Constructing a competitive endogenous RNA network of EndMT-related atherosclerosis through weighted gene co-expression network analysis

Yubiao Wu^1†^, Yawei Li^1†^, Xiude Qin^2†^, Jinchao Gu^1^, Aijun Liu^1^, Jiahui Cao^1*^

*** Correspondence:** Jiahui Cao, [caojiahui@gzucm.edu.cn](mailto:caojiahui@gzucm.edu.cn).

# Supplementary Figures and Tables

For more information on Supplementary Material and for details on the different file types accepted, please see [here](https://www.frontiersin.org/guidelines/author-guidelines#supplementary-material).

## Supplementary Figures

**
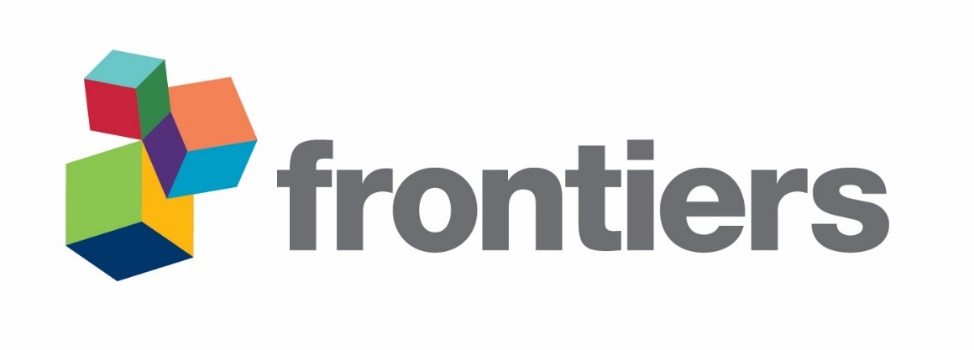
**

**Supplementary Figure 1.** Clustering dendrogram of samples based on their Euclidean distance. (A) shows the clustering dendrogram before removal of outlier samples. (B) shows the clustering dendrogram after removal of outlier samples.

**Supplementary Figure 2.** Construction of weighted co-expression network and module analysis. (A) Selection process for soft thresholding; (B) Cluster dendrogram. The colored blocks represent distinct gene modules, each comprising genes with similar expression patterns. The gray blocks contain genes that failed to be clustered. After three rounds of iterative screening, the gray blocks were removed. (C) Adjacency heatmap of module eigengenes.

**Supplementary Figure 3.**PPI network of genes present in the intersection between the black module (A), blue module (B), and downregulated genes in the EndMT dataset.

**Supplementary Figure 4.** Enrichment analysis of the turquoise module. (A) Results of KEGG enrichment analysis, (B) Enriched biological processes (BP), (C) Enriched cellular components (CC), and (D) Enriched molecular functions (MF).

**Supplementary Figure 5.** Expression levels of hub genes in atherosclerosis and EndMT validation datasets. (A) Expression levels of hub genes in GSE28829, containing 16 advanced carotid plaques and 13 early carotid plaques. (B) Expression levels of hub genes in GSE97210, containing 3 advanced plaques and 3 normal vessel tissues.(C) Expression levels of hub genes in GSE43292, containing 32 carotid plaques and 32 normal vessel tissues.(D) Expression levels of hub genes in carotid artery samples from GSE100927, containing 21 carotid plaques and 10 normal carotid arteries, where CA_plaque indicates carotid plaque.(E) Expression levels of hub genes in femoral artery samples from GSE100927, containing 25 femoral plaques and 10 normal femoral arteries, where FA_plaque indicates femoral plaque.(F) Expression levels of hub genes in infrapopliteal artery samples from GSE100927, containing 11 infrapopliteal plaques and 8 normal infrapopliteal arteries, where IPA_plaque indicates infrapopliteal plaque. Expression levels of hub genes in HUVECs after 7 days of TGF-β2 induction from GSE118446(G), in HPAECs after 7 days of combined TGF-β2 and IL-1β induction from GSE118446 (H), in HUVECs after 8 hours of TGF-β3 induction from GSE65545 (I), in HUVECs after 5 days of TGF-β2 and H2O2 induction from GSE56309(J), in HUVECs after 7 days of IL-1β induction from GSE118446 (K), in HUVECs after 12 hours of TNFα induction from GSE203505 (L), in HUVECs after 12 hours of TNFα induction from GSE184512(M), in HUVECs after 24 hours of TNFα induction from GSE203505(N), and in HUVECs after 24 hours of TNFα induction from GSE188403(O).*_P_<0.05, **_P_<0.01, ***_P_<0.001.

**Supplementary Figure 6.** The locations of the hub genes in the Toll-like receptor signaling pathway. The hub genes are marked in pink, while other genes in the pathway are marked in green.

**Supplementary Figure 7.** Expression of EndMT markers in the EndMT validation datasets.

**Supplementary Figure 8.** Validation of the miRNAs and lncRNAs in atherosclerosis and EndMT validation datasets. Heatmaps display the logFC of miRNAs (A) and lncRNAs (B) across the validation datasets. Red indicates up-regulation, green indicates down-regulation, black indicates the miRNA or lncRNA is not present in that particular dataset.

**Supplementary Figure 9.** The analysis rationale for elucidating the ceRNA network mechanism through which DSY regulates EndMT to treat atherosclerosis. Hub genes: Key genes that can simultaneously regulate both EndMT and AS. miRNA1: miRNAs that were downregulated in both AS and EndMT as described above. lncRNA1: lncRNAs that were upregulated in both AS and EndMT as presented above. Hub1 genes: The hub genes regulated by miRNA1 and lncRNA1. Hub2 genes: Key genes regulated by DSY in the EndMT dataset. mRNA1: mRNAs of Hub1 genes. mRNA2: mRNAs of Hub2 genes. miRNA2: miRNAs that can regulate mRNA2. miRNA3: Intersection of miRNA1 and miRNA2. lncRNA3: lncRNA1 after further screening. Solid lines represent content that was previously validated, and dashed lines represent hypothetical relationships that need further validation in follow-up studies.

**Supplementary Figure 10.** The active component of DSY-mRNA2-miRNA3 network.

**Supplementary Figure 11.** Expression level of lncRNA in the atherosclerosis and EndMT validation datasets.

**Supplementary Figure 12.** Molecular docking results of the top three DSY active compounds ranked by binding affinity with lncRNA.

**Supplementary Figure 13.**  Network of distinct active ingredient-lncRNA-miRNA-mRNA.

**Supplementary Figure 14.** Heatmap of docking binding energies between Hub1 genes and active components of DSY.

## Supplementary Tables

| model color | number of mRNAs | most significant term | p.adjusted |
| --- | --- | --- | --- |
| black | 103 | GO:0009952 anterior/posterior pattern specification | 7.26E-15 |
| blue | 628 | GO:0043292 contractile fiber | 4.04E-22 |
| brown | 226 | GO:0008380 RNA splicing | 4.91E-02 |
| red | 110 | Hsa03008 ribosome biogenesis in eukaryotes | 1.05E-03 |
| turquoise | 4869 | GO:0001819 positive regulation of cytokine production | 2.04E-30 |
| green | 144 | No enrichment of significant term | — |
| yellow | 150 | No enrichment of significant term | — |

**Supplementary table 1.** Enrichment analysis of gene ontology biological processes for the modules

**Supplementary table 2.** Overview of atherosclerosis validation datasets

| **Dataset name** | **Grouping status (number of cases)** | **Category** |
| --- | --- | --- |
| **GSE97210** | Advanced atherosclerotic plaque in the common carotid artery (3)  Normal abdominal aortic intima (3) | Atherosclerosis validation set |
| **GSE28829** | Advanced carotid plaque (16)  Early carotid plaque (13) | Atherosclerosis validation set |
| **GSE43292** | Carotid plaque (32)  Normal tissue surrounding plaque (32) | Atherosclerosis validation set |
| **GSE100927** | Carotid plaque in men (21)  Normal carotid arteries in men (10) | Atherosclerosis validation set |
| **GSE100927** | Femoral artery plaque in men (25)  Normal femoral artery in men (10) | Atherosclerosis validation set |
| **GSE100927** | Subpopliteal artery plaque in men (11)  Normal subpopliteal artery in men (8) | Atherosclerosis validation set |
| **GSE118446** | HUVEC_TGF-β2_7D(3)  HUVEC_CON（3） | EndMT validation set |
| **GSE118446** | HUVEC_IL-1β_7D(3)  HUVEC_CON（3） | EndMT validation set |
| **GSE118446** | HPAEC_TGF-β2+IL-1β_7D（3）  HPAEC_CON(3) | EndMT validation set |
| **GSE56309** | HUVEC_TGF-β2+H2O2_5D(4)  HUVEC_CON(3) | EndMT validation set |
| **GSE65445** | VEC_TGF-β3_8H（3)  VEC_CON(3) | EndMT validation set |
| **GSE203505** | HUVEC_TNFα_24H(2)  HUVEC_CON_24H(2) | EndMT validation set |
| **GSE203305** | HUVEC_TNFα_12H(2)  HUVEC_CON_12H(2) | EndMT validation set |
| **GSE184512** | HUVEC_TNFα_12H(2)  HUVEC_CON_12H(2) | EndMT validation set |
| **GSE144803** | HUVEC_TNFα_24H(39)  HUVEC_CON_24H(39) | EndMT validation set |

**Supplementary table 3.** Overview of EndMT validation datasets

| **Dataset name** | **Grouping status (number of cases)** | **Category** |
| --- | --- | --- |
| **GSE137580** | HAEC oxLDL processing 48H (3)  HA con(3) | miRNA validation set |
| **GSE89858** | Ascending aorta of ApoBTM2Sgy/Ldltm1Her mice modeled with high fat for 30 weeks (2)  Ascending aorta of ApoBTM2Sgy/Ldltm1Her mice fed a normal diet for 30 weeks (3) | miRNA validation set |
| **GSE26555** | Carotid arteries of APOE-/- mice treated with high-fat diet for 6 weeks and partially ligated (3)  Carotid arteries of APOE-/- mice without treatment on high-fat diet for 6 weeks (3) | miRNA validation set |
| **GSE97210** | Advanced atherosclerotic plaque in the common carotid artery (3)  Normal abdominal aortic intima (3) | LncRNA validation set |
| **GSE207252** | carotid plaque (2)  Normal vascular intima tissue (2) | LncRNA validation set |
| **GSE83112** | Aorta of APOE-/- mice induced by high-fat diet for 8 weeks (3)  Aortas of APOE-/- mice treated with normal diet for 8 weeks (3) | LncRNA validation set |
| **GSE118446** | HUVEC_TGFβ2_7D(3)  HUVEC_CON（3） | LncRNA validation set |
| **GSE118446** | HUVEC_IL1B_7D(3)  HUVEC_CON（3） | LncRNA validation set |
| **GSE118446** | HUVEC_TGFβ2+IL1B_7D（3）  HUVEC_CON(3) | LncRNA validation set |
| **GSE118446** | HPAEC_TGFβ2+IL1B_7D(3)  HPAEC_CON(3) | LncRNA validation set |
| **GSE173719** | Peripheral blood exosomes from patients with large artery atherosclerosis and stroke (5)  Healthy control peripheral blood exosomes (5) | circRNA validation set |
| **GSE161913** | Peripheral blood mononuclear cells from patients with acute ischemia due to large artery atherosclerosis (5)  Healthy control peripheral blood mononuclear cells (4) | circRNA validation set |
| **GSE133269** | Aorta of APOE-/- mice induced by high-fat diet for 3 months (2)  Normal mouse aorta (2) | circRNA validation set |

**Supplementary table 4** Molecular docking between lncRNA and active components in DSY

| **lncRNA** | **Active ingredients** | **Binding energy（kcal/mol）** | **Hydrogen bonding interaction bases** |
| --- | --- | --- | --- |
| **AC003092.1** | Salvianolic acid B | -11.4 | U60、C80、A101、A102、U113 |
| **AC003092.1** | Tanshinone I | -10.9 | A101、A102 |
| **AC003092.1** | Polydatin | -10.5 | A420、U440、U450  U460、C580、A600、A610 |
| **AC003092.1** | Morphine | -10.2 | A470、C570、C580 |
| **AC003092.1** | Luteolin | -10.2 | A560、A600、A610、C580、U590 |
| **AC003092.1** | Quercetin | -10.1 | A420、U460、U590、A600、A610、 |
| **AC003092.1** | Apigenin | -9.6 | A470、C580、U590、U450 |
| **AC003092.1** | Vitamin C | -7.1 | G193、U113、A101、U194 |
| **AC003092.1** | Palmitic acid | -5.7 | C570、C580、U460 |
| **KRTAP5-AS1** | Rhein | -10.7 | A767、C775、A807、C808、G824 |
| **KRTAP5-AS1** | Polydatin | -10.7 | A367、U415、A417、G422、G444、A445 |
| **KRTAP5-AS1** | Quercetin | -10.6 | U836、G837、A839、U926 |
| **KRTAP5-AS1** | Tanshinone Ⅰ | -10.3 | U866 |
| **KRTAP5-AS1** | Cryptomeria | -9.6 | #N/A |
| **KRTAP5-AS1** | Aloe emodin | -9.5 | G1297 |
| **KRTAP5-AS1** | Protocatechuic acid | -7.6 | A767、C808 |
| **KRTAP5-AS1** | Eugenol | -7.2 | #N/A |
| **KRTAP5-AS1** | Vitamin C | -7.0 | A634、G635、G636、C565、C566、U567 |
| **KRTAP5-AS1** | Eucalyptol | -5.7 | #N/A |
| **KRTAP5-AS1** | Palmitic acid | -5.2 | A779、C810、G823、G824、 |
| **MIR137HG** | Ellagic acid | -12.6 | G1537、G1538、A1540、U1546、A1548、 |
| **MIR137HG** | Salvianolic acid B | -11.4 | A215、C216、U276、A277  G284、C990、G1518、U1519 |
| **MIR137HG** | Rhein | -11.3 | G284、C990、U1517 |
| **MIR137HG** | Tanshinone I | -11.0 | G284 |
| **MIR137HG** | Polydatin | -11.0 | U261、G262、G1537 |
| **MIR137HG** | Quercetin | -10.9 | C280、C2128、A2129 |
| **MIR137HG** | Aloe emodin | -10.7 | G284、U1510、U1511、G1518 |
| **MIR137HG** | Kaempferol | -10.6 | C2131 |
| **MIR137HG** | Luteolin | -10.6 | G287、G1518 |
| **MIR137HG** | Morphine | -10.3 | A260 |
| **MIR137HG** | Apigenin | -10.1 | U285、U1517 |
| **MIR137HG** | Vitamin C | -7.7 | U1529、1533、C2131 |
| **MIR137HG** | Protocatechuic acid | -7.5 | C990 |
| **MIR137HG** | Eugenol | -6.7 | G159、U1510 |
| **MIR137HG** | Eucalyptol | -5.9 | A2429 |
| **MIR137HG** | Palmitic acid | -5.6 | C2156、C2157、C2166 |
| **MIR155HG** | Salvianolic acid L | -12.0 | G775、A777、A1226、G1227、U1229、U1230 |
| **MIR155HG** | Salvianolic acid B | -11.8 | G776、U778、A1222、  U1225、A1226、A1228、U1229 |
| **MIR155HG** | Polydatin | -11.3 | A777、U778、U781、A1226、A1228 |
| **MIR155HG** | Tanshinone I | -11.0 | C782 |
| **MIR155HG** | Tanshinone IIA | -10.9 | C782 |
| **MIR155HG** | Morphine | -10.7 | G1227 |
| **MIR155HG** | Ellagic acid | -10.6 | U1225、A1228 |
| **MIR155HG** | Rhein | -10.5 | U779、G780、C782、A1222 |
| **MIR155HG** | Cryptomeria | -10.4 | G775、A777、A1232 |
| **MIR155HG** | Quercetin | -10.1 | A777、U778、C782、G1227 |
| **MIR155HG** | Luteolin | -10.1 | A777、U1225、G1227 |
| **MIR155HG** | aloe emodin | -10.0 | C782、A1222、A1224 |
| **MIR155HG** | Kaempferol | -9.8 | U1225 |
| **MIR155HG** | Apigenin | -9.8 | A1226、U1230 |
| **MIR155HG** | Butylated hydroxytoluene | -7.4 | #N/A |
| **MIR155HG** | Vitamin C | -6.9 | U774、A1231、A1232 |
| **MIR155HG** | Protocatechuic acid | -6.6 | A1226、A1227、A1228 |

**Supplementary table 5** The top 10 hub genes

| **Hub gene** | **Degree** |
| --- | --- |
| **TNF** | 244 |
| **IL1B** | 184 |
| **STAT1** | 164 |
| **TLR4** | 150 |
| **TLR2** | 128 |
| **IRF7** | 116 |
| **CXCL10** | 112 |
| **CD44** | 106 |
| **MYD88** | 106 |
| **CD68** | 100 |
