# Supplementary figures and images for: Constructing a competitive endogenous RNA network of EndMT-related atherosclerosis through weighted gene co-expression network analysis
Source: Front Cardiovasc Med. 2024 Jan 10;10:1322252. doi: 10.3389/fcvm.2023.1322252 (PMC10806165; doi:10.3389/fcvm.2023.1322252)

A

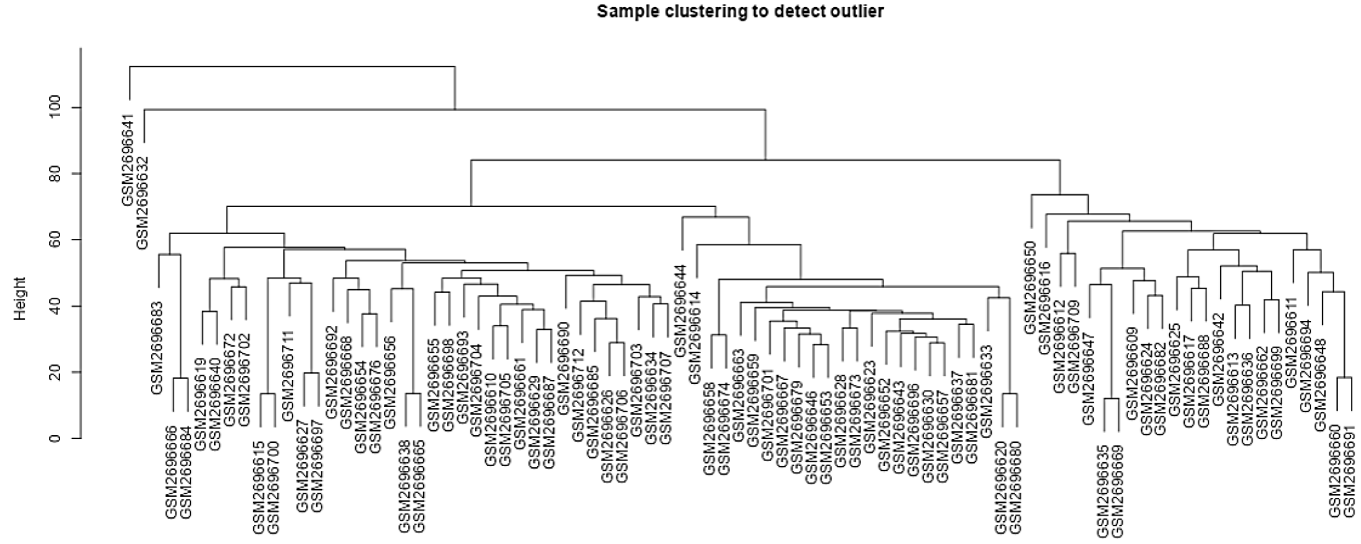

B

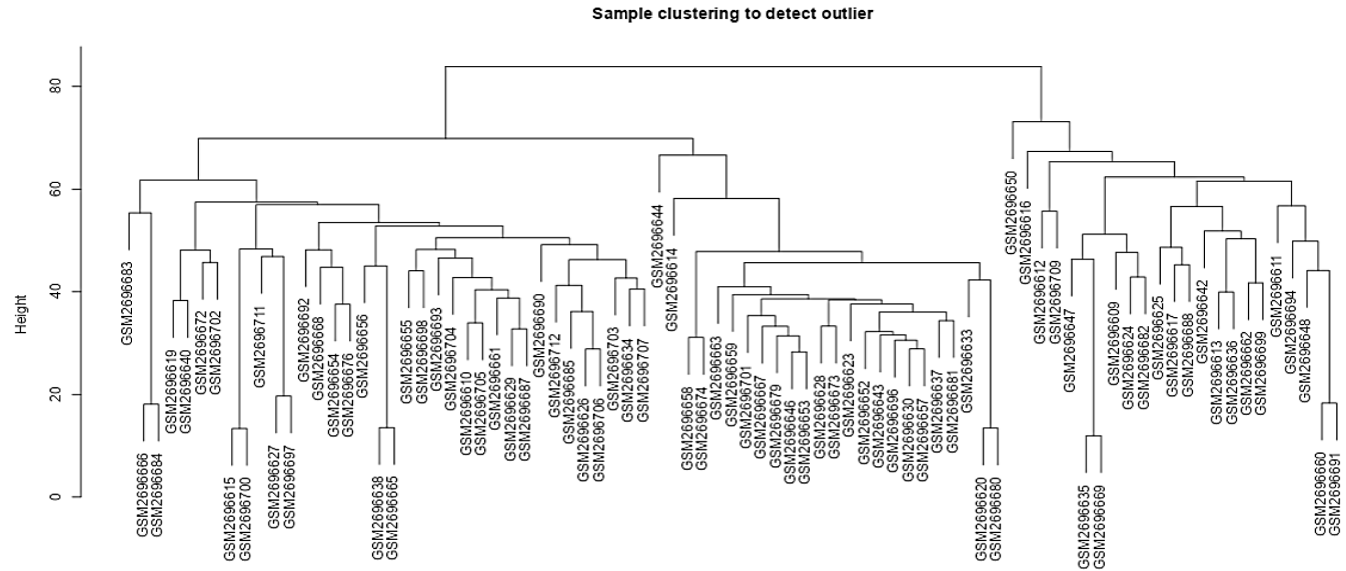

Supplement: Supplementary file 2 [file Image1.pdf]

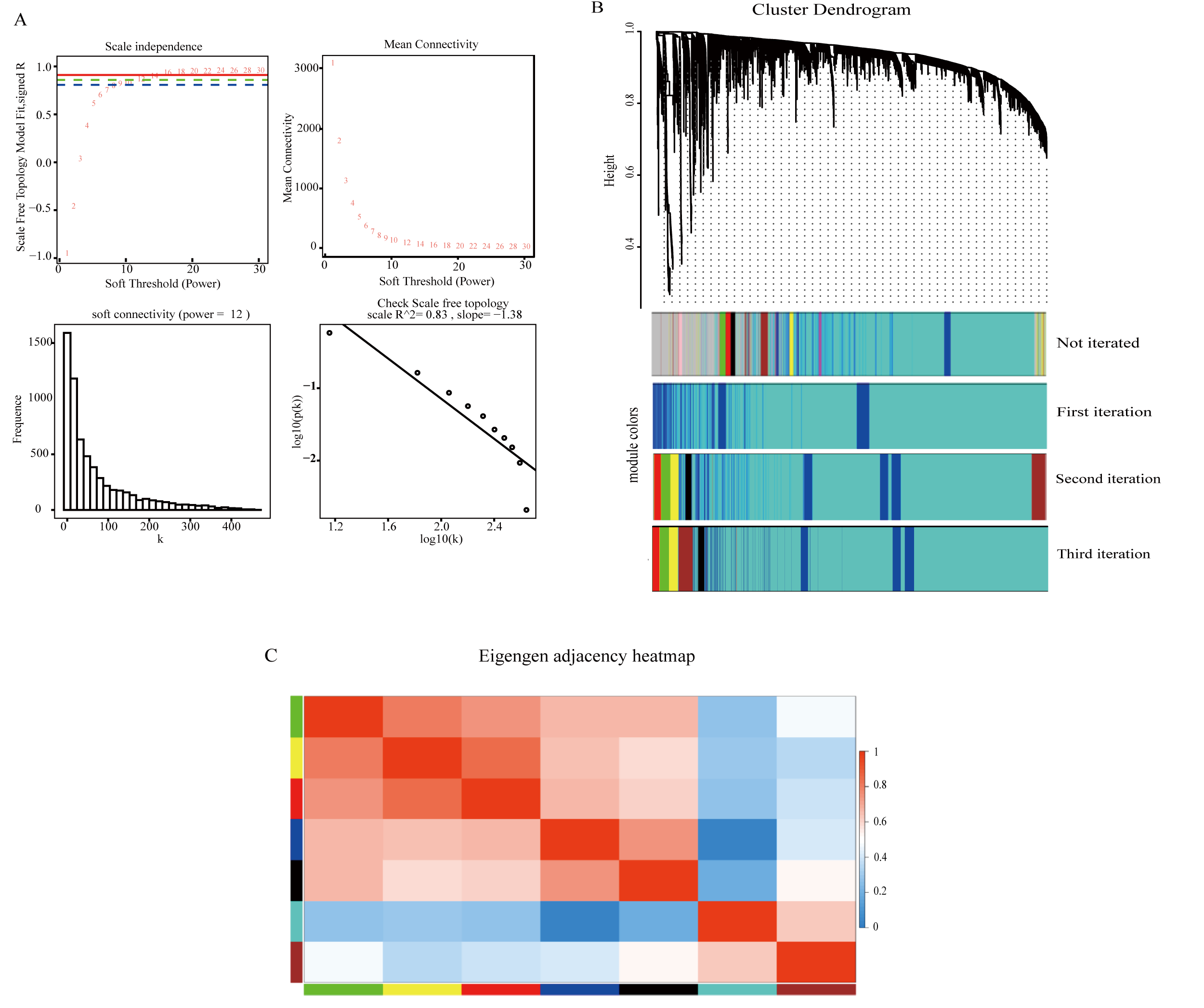

Supplement: Supplementary file 3 [file Image2.png]

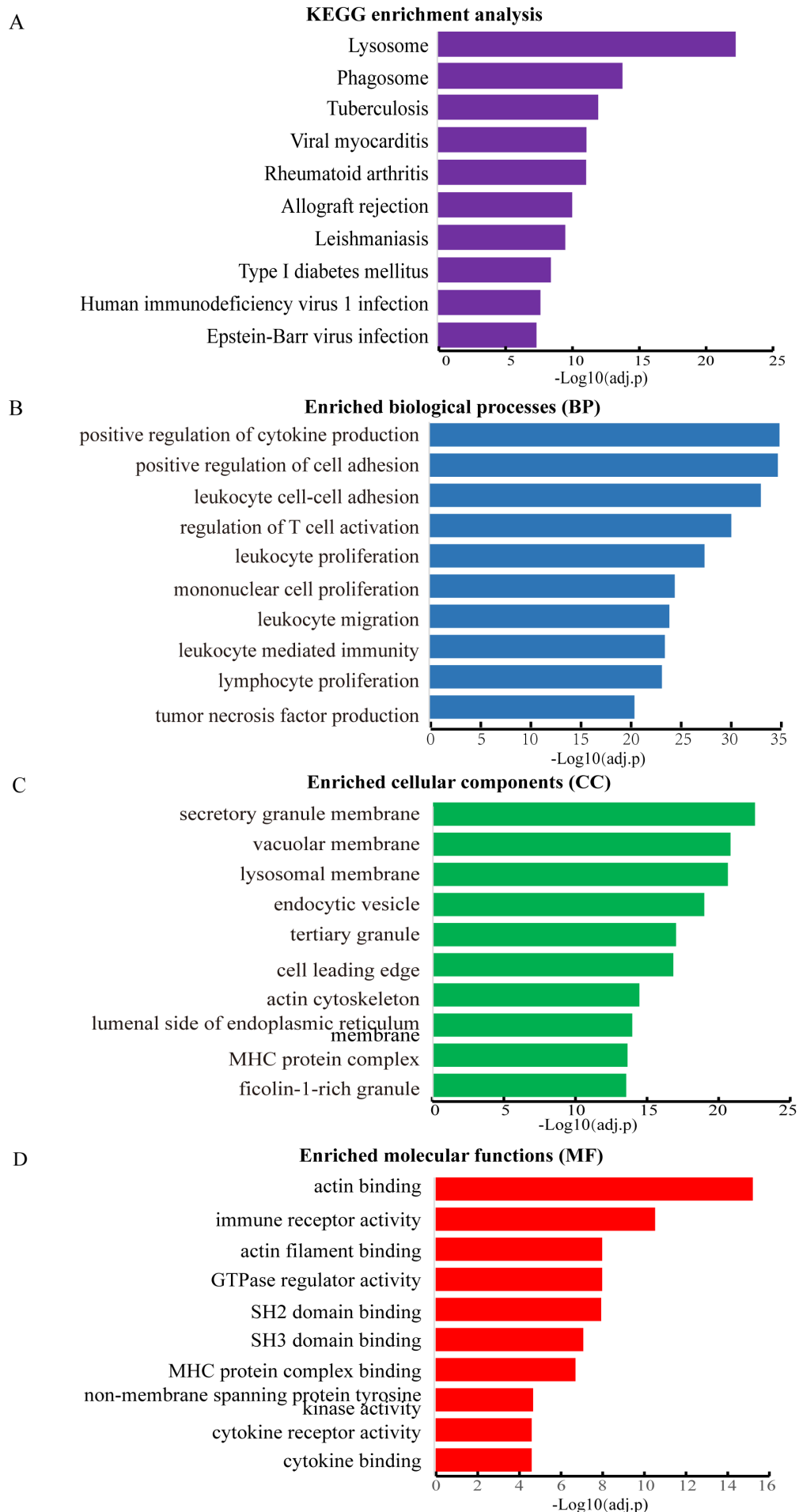

Supplement: Supplementary file 4 [file Image3.pdf]

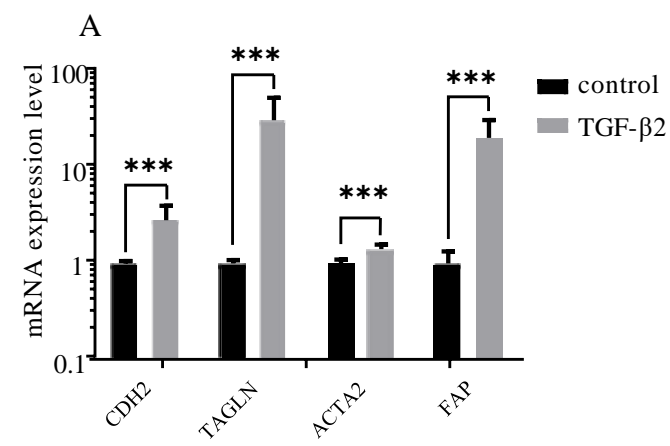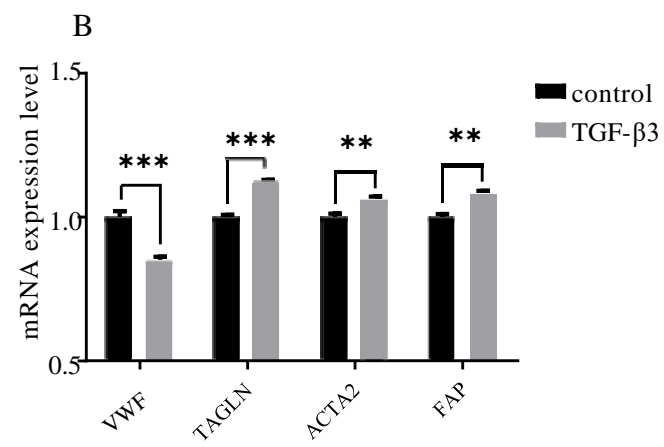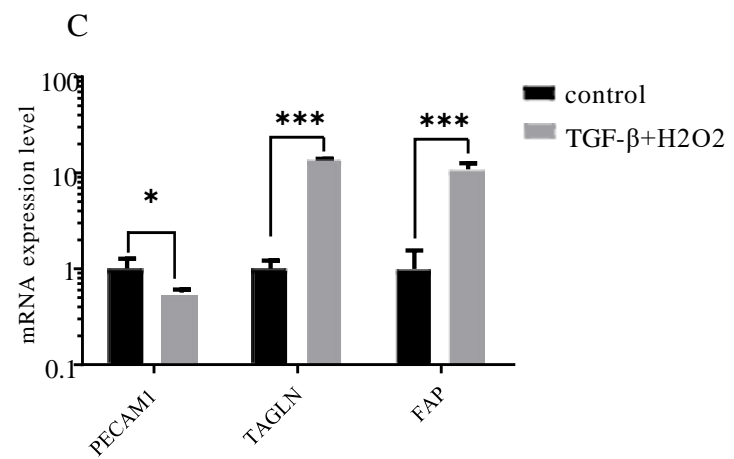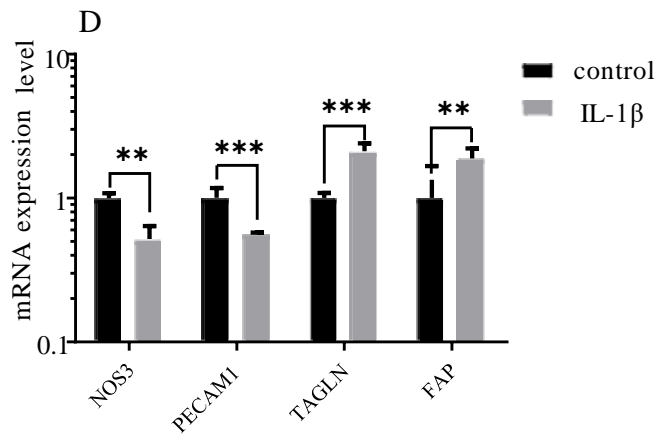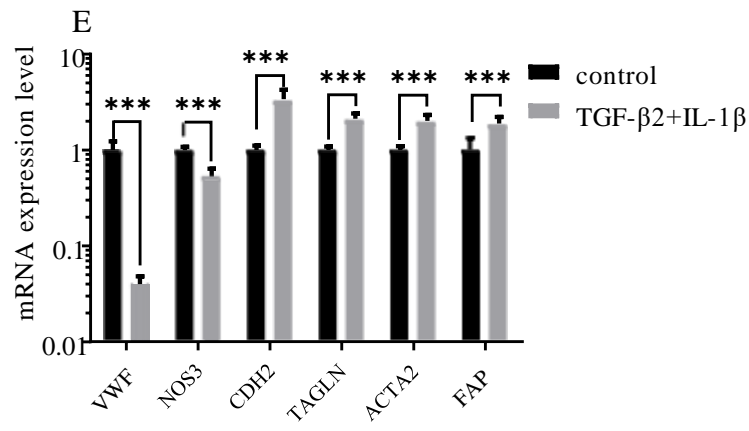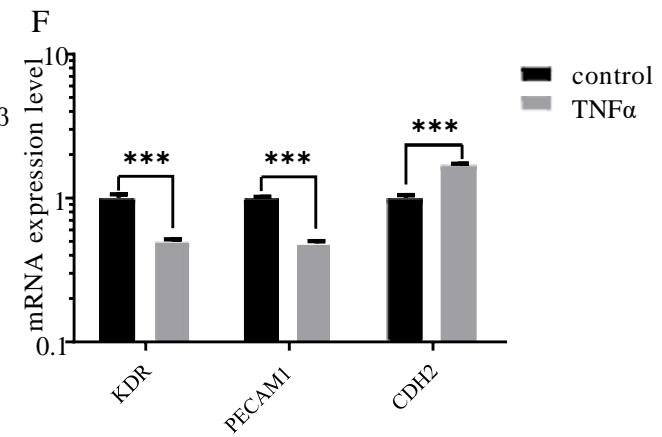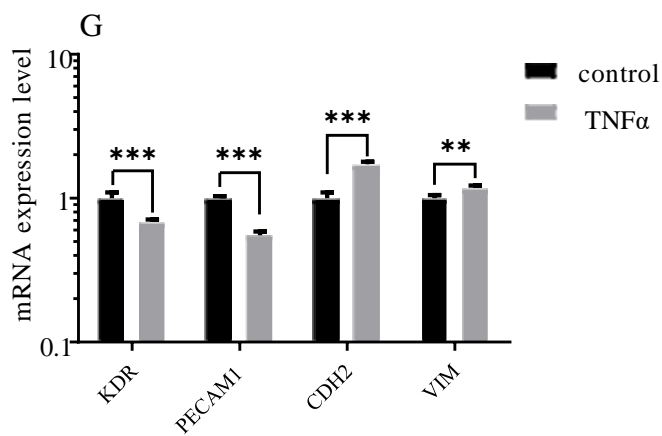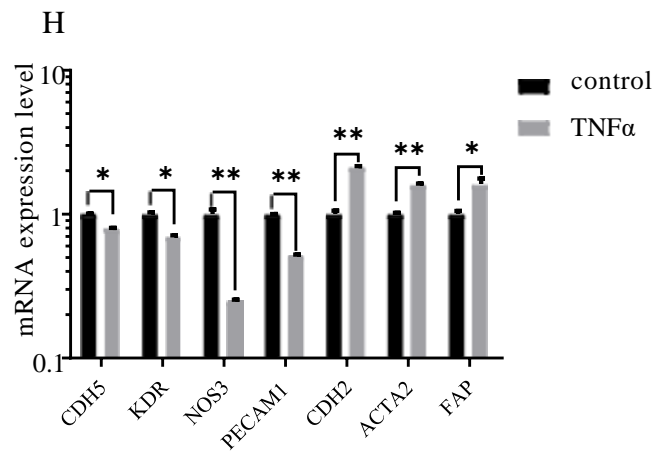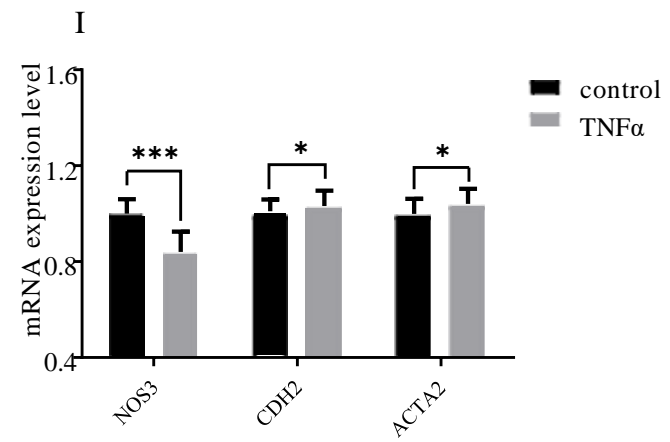

Supplement: Supplementary file 6 [file Image5.pdf]

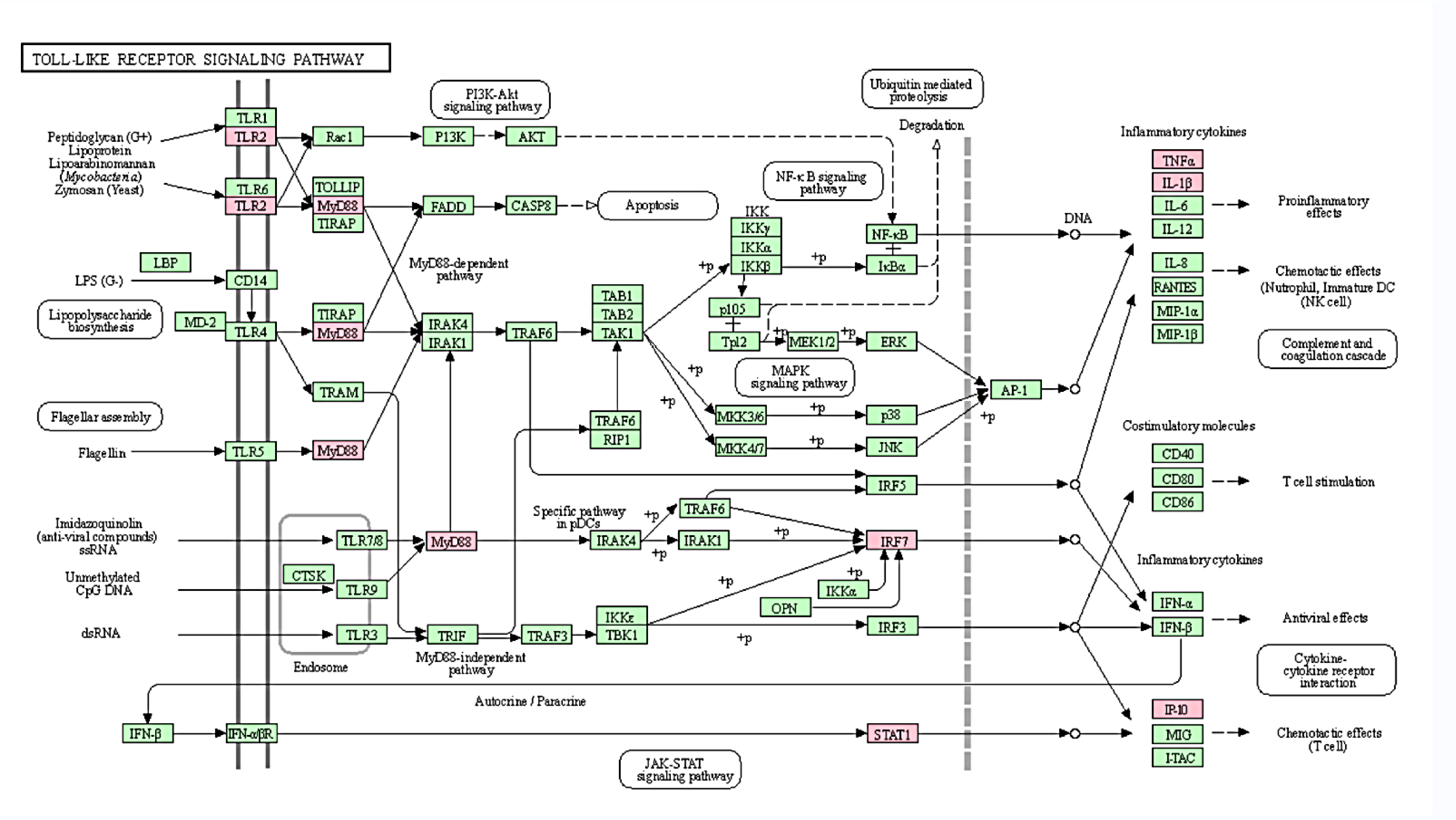

Supplement: Supplementary file 7 [file Image6.png]

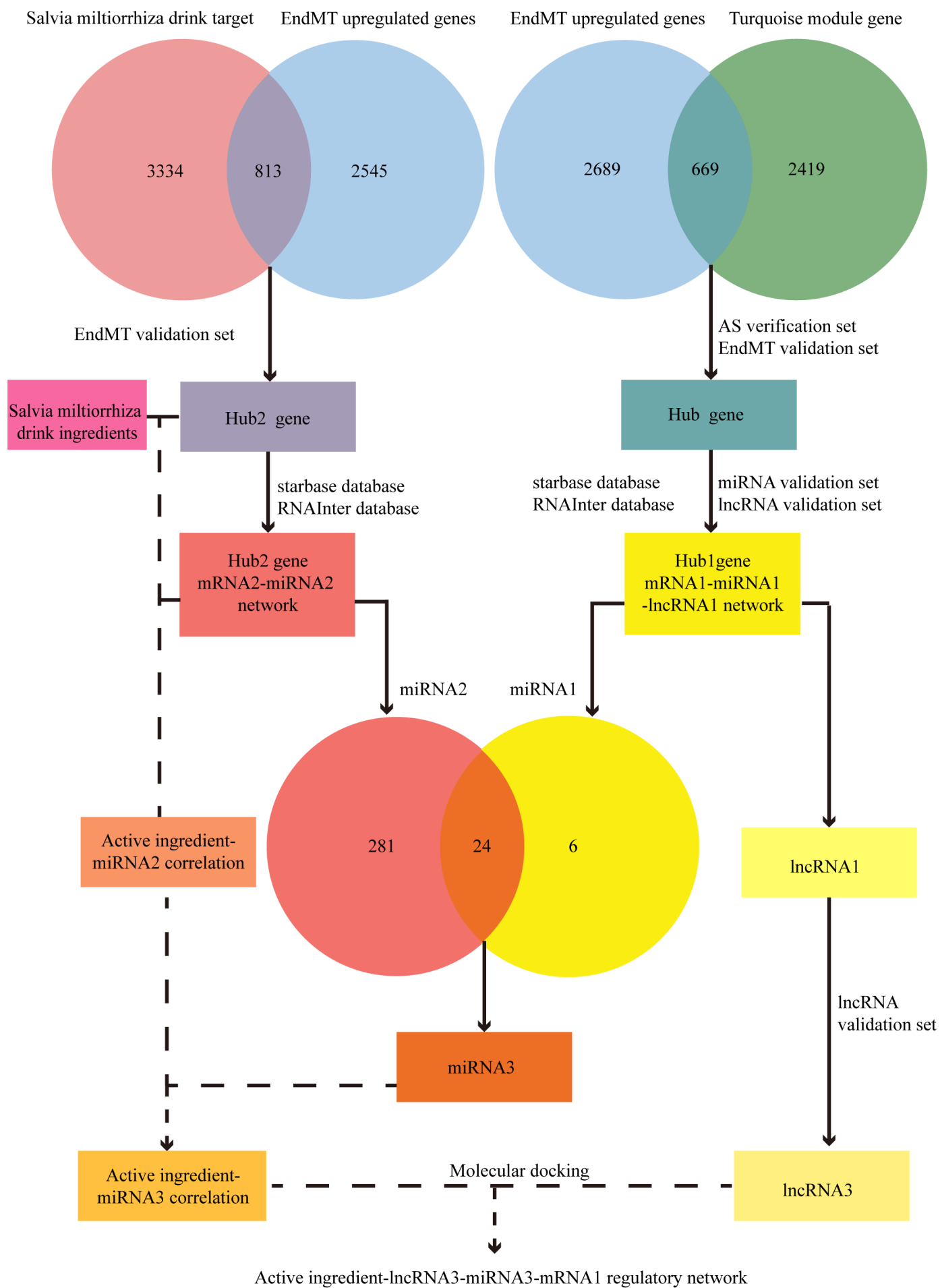

Supplement: Supplementary file 8 [file Image7.pdf]

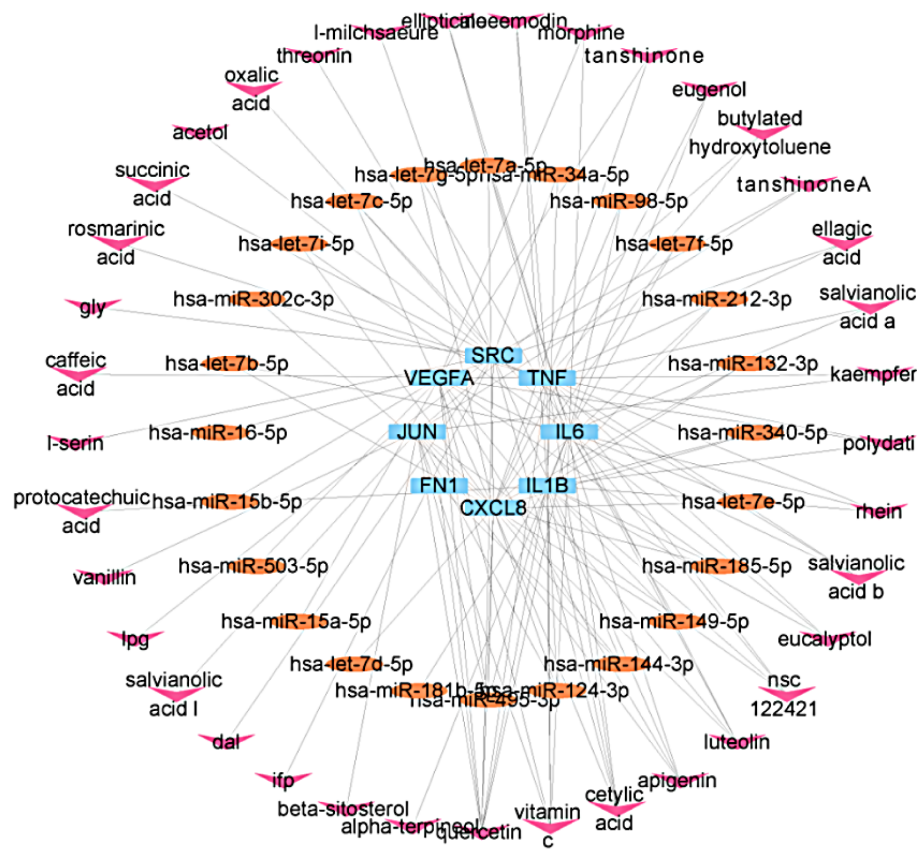

Supplement: Supplementary file 9 [file Image8.pdf]

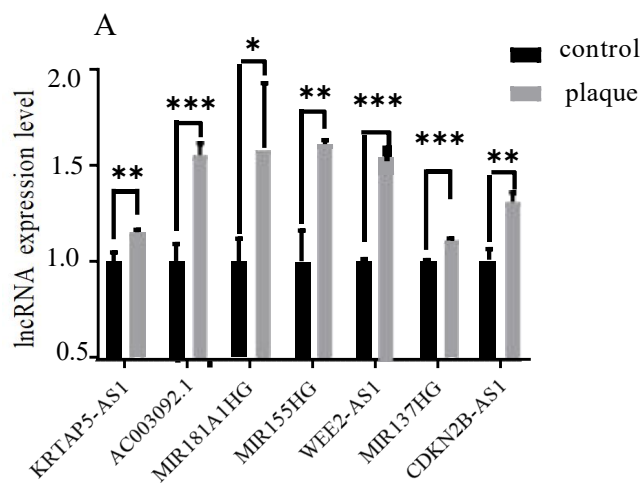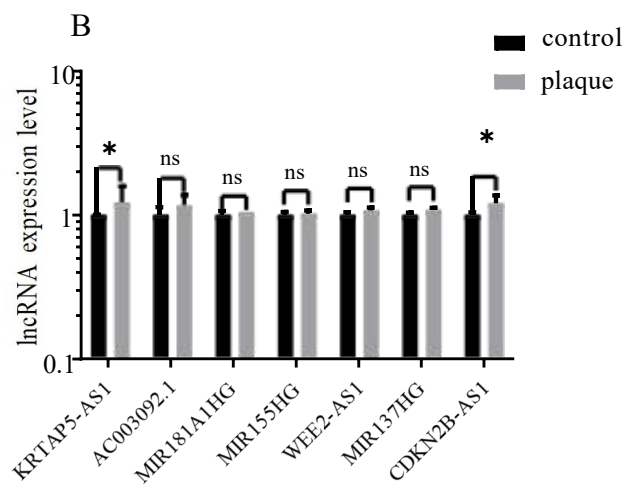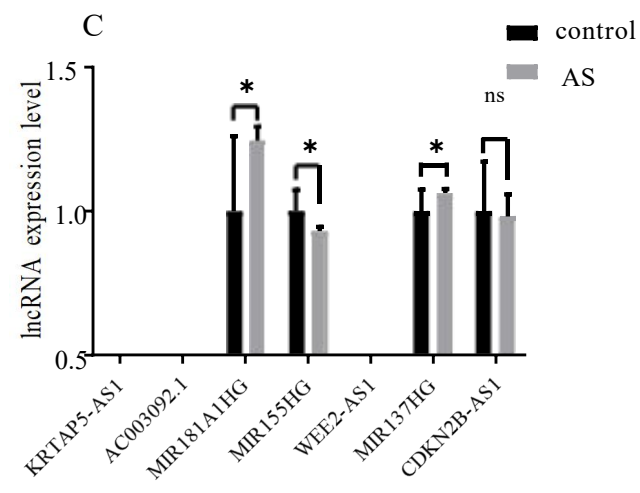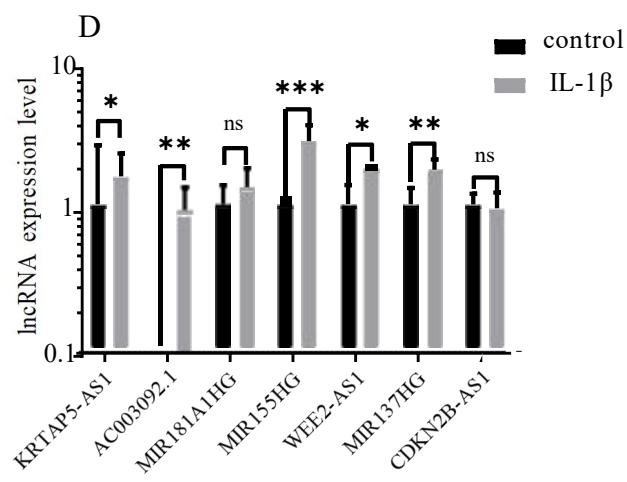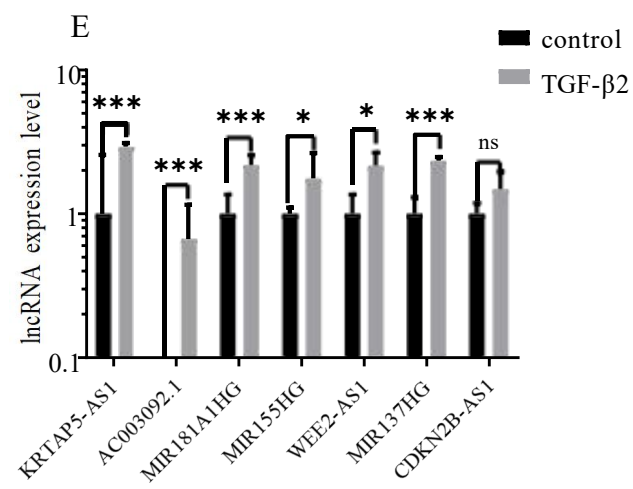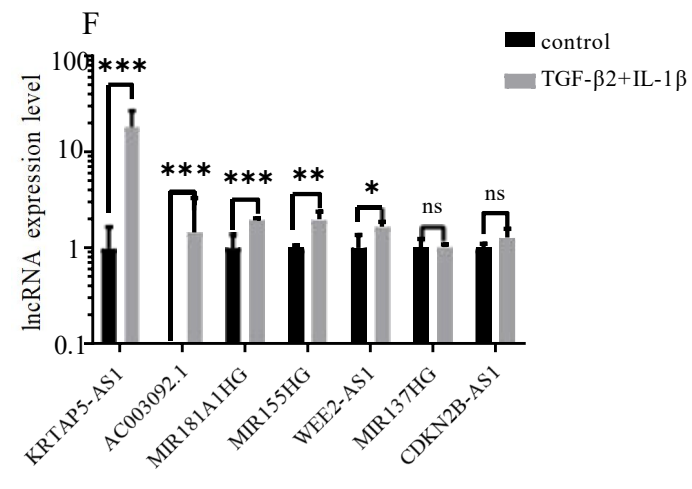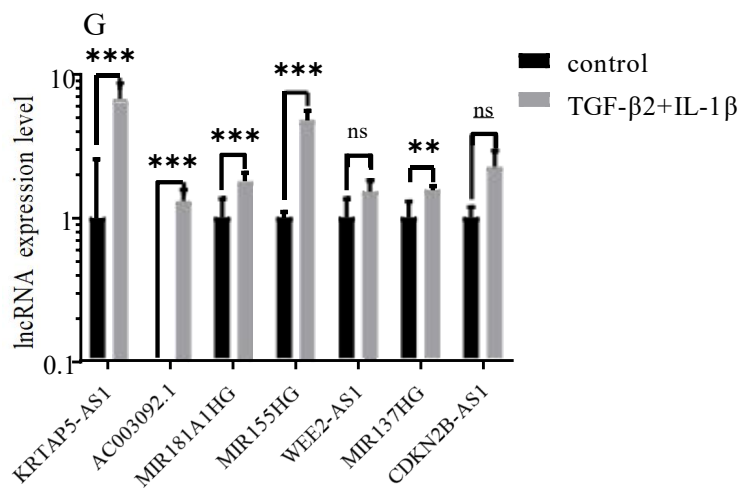

Supplement: Supplementary file 10 [file Image9.pdf]

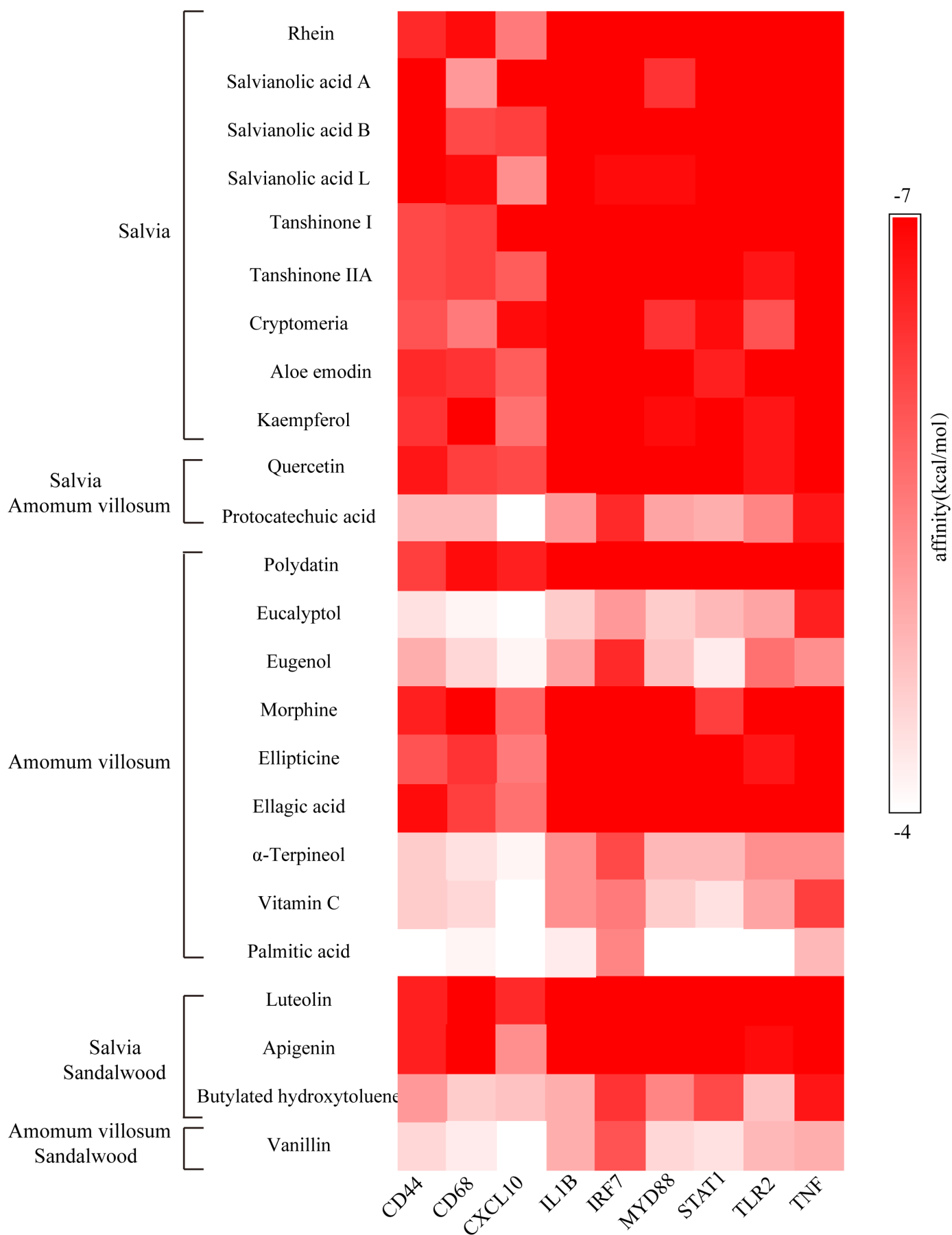

Supplement: Supplementary file 13 [file Image12.pdf]
