# Supplementary material for: Constructing a competitive endogenous RNA network of EndMT-related atherosclerosis through weighted gene co-expression network analysis
Source: Front Cardiovasc Med. 2024 Jan 10;10:1322252. doi: 10.3389/fcvm.2023.1322252 (PMC10806165; doi:10.3389/fcvm.2023.1322252)
Supplement: Supplementary file 11 [file Image10.pdf]

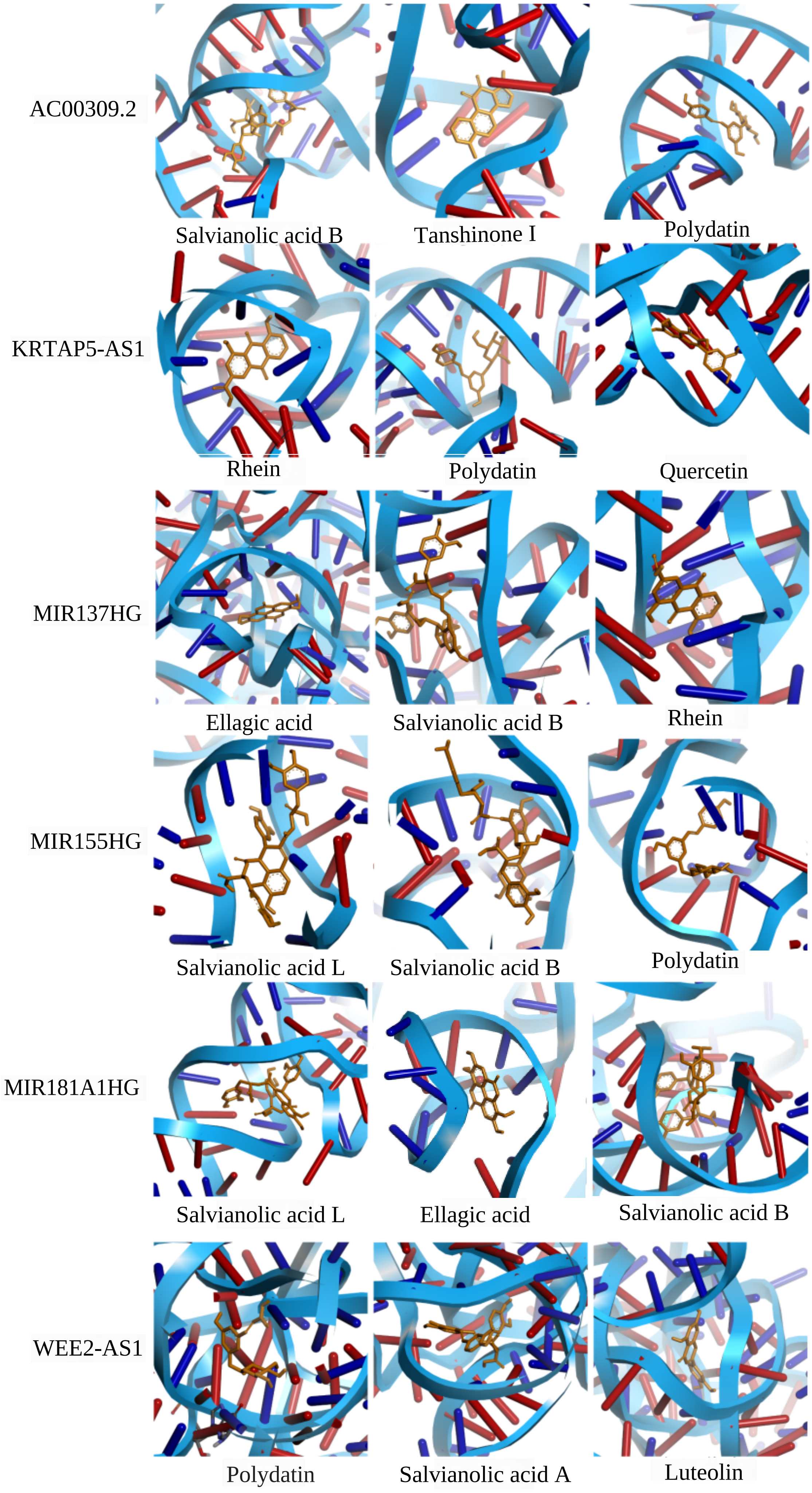
AC00309.2
Salvianolic acid B
Tanshinone I
Polydatin
KRTAP5-AS1
Rhein
Polydatin
Quercetin
MIR137HG
Ellagic acid
Salvianolic acid B
Rhein
MIR155HG
Salvianolic acid L
Salvianolic acid B
Polydatin
MIR181A1HG
Salvianolic acid L
Ellagic acid
Salvianolic acid B
WEE2-AS1
Polydatin
Salvianolic acid A
Luteolin
