# Supplementary material for: Constructing a competitive endogenous RNA network of EndMT-related atherosclerosis through weighted gene co-expression network analysis
Source: Front Cardiovasc Med. 2024 Jan 10;10:1322252. doi: 10.3389/fcvm.2023.1322252 (PMC10806165; doi:10.3389/fcvm.2023.1322252)
Supplement: Supplementary file 12 [file Image11.pdf]

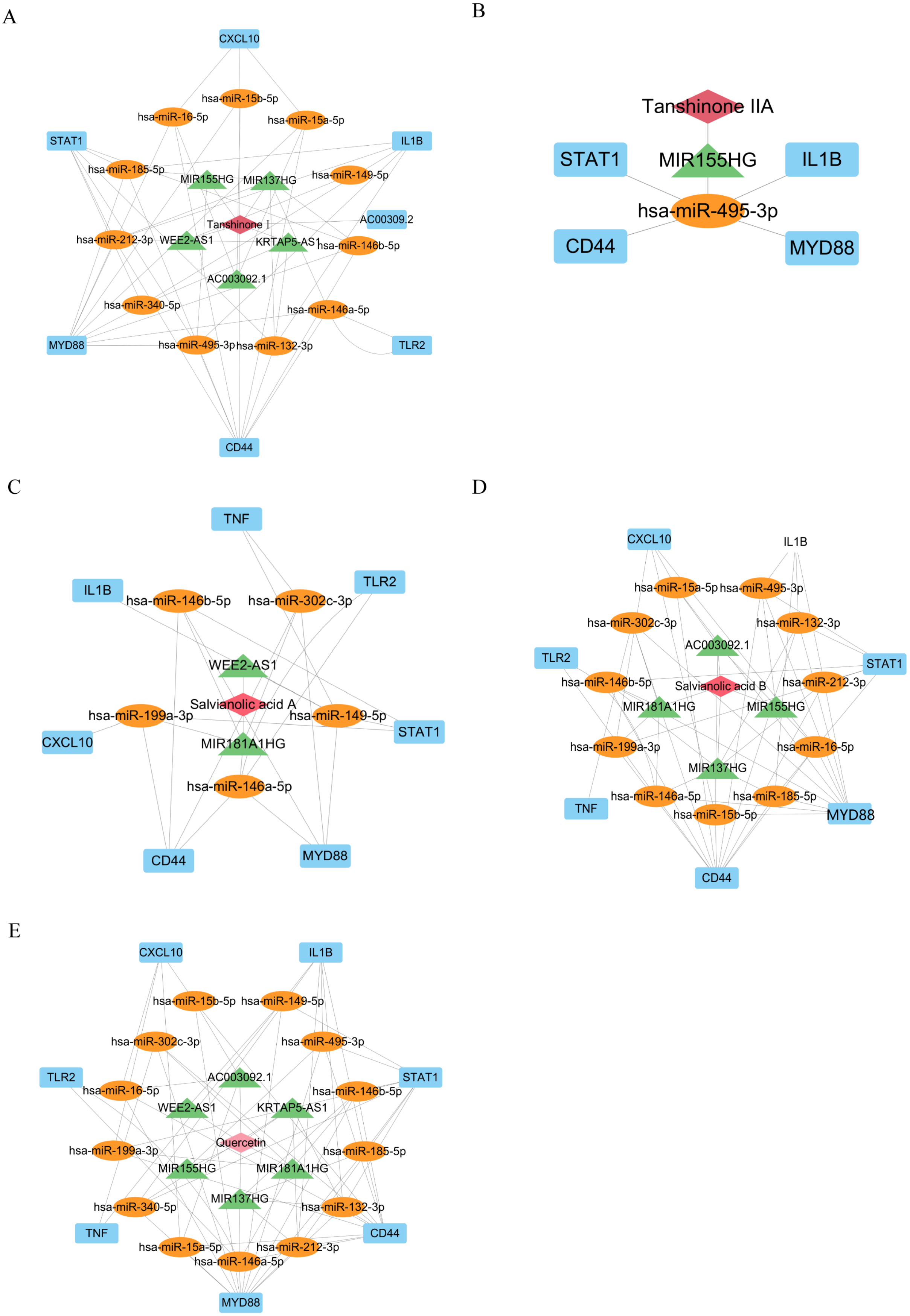
A
CXCL10
hsa-miR-15b-5p
hsa-miR-16-5p
hsa-miR-15a-5p
STAT1
IL1B
hsa-miR-185-5p
MIR155HG
MIR137HG
hsa-miR-149-5p
Tanshinone I
AC00309.2
hsa-miR-212-3p
WEE2-AS1
KRTAP5-AS1
hsa-miR-146b-5p
AC003092.1
hsa-miR-340-5p
hsa-miR-146a-5p
MYD88
hsa-miR-495-3p
hsa-miR-132-3p
TLR2
CD44
B
Tanshinone IIA
STAT1
MIR155HG
IL1B
hsa-miR-495-3p
CD44
MYD88
C
TNF
IL1B
TLR2
hsa-miR-146b-5p
hsa-miR-302c-3p
WEE2-AS1
Salvianolic acid A
hsa-miR-199a-3p
hsa-miR-149-5p
CXCL10
STAT1
MIR181A1HG
hsa-miR-146a-5p
CD44
MYD88
D
CXCL10
IL1B
hsa-miR-15a-5p
hsa-miR-495-3p
hsa-miR-302c-3p
hsa-miR-132-3p
AC003092.1
TLR2
STAT1
hsa-miR-146b-5p
Salvianolic acid B
hsa-miR-212-3p
MIR181A1HG
MIR155HG
hsa-miR-199a-3p
hsa-miR-16-5p
MIR137HG
TNF
hsa-miR-146a-5p
hsa-miR-185-5p
hsa-miR-15b-5p
MYD88
CD44
E
CXCL10
IL1B
hsa-miR-15b-5p
hsa-miR-149-5p
hsa-miR-302c-3p
hsa-miR-495-3p
TLR2
AC003092.1
STAT1
hsa-miR-16-5p
hsa-miR-146b-5p
WEE2-AS1
KRTAP5-AS1
Quercetin
hsa-miR-199a-3p
hsa-miR-185-5p
MIR155HG
MIR181A1HG
MIR137HG
hsa-miR-340-5p
hsa-miR-132-3p
TNF
CD44
hsa-miR-15a-5p
hsa-miR-212-3p
hsa-miR-146a-5p
MYD88
