# Supplementary material for: Constructing a competitive endogenous RNA network of EndMT-related atherosclerosis through weighted gene co-expression network analysis
Source: Front Cardiovasc Med. 2024 Jan 10;10:1322252. doi: 10.3389/fcvm.2023.1322252 (PMC10806165; doi:10.3389/fcvm.2023.1322252)
Supplement: Supplementary file 5 [file Image4.pdf]

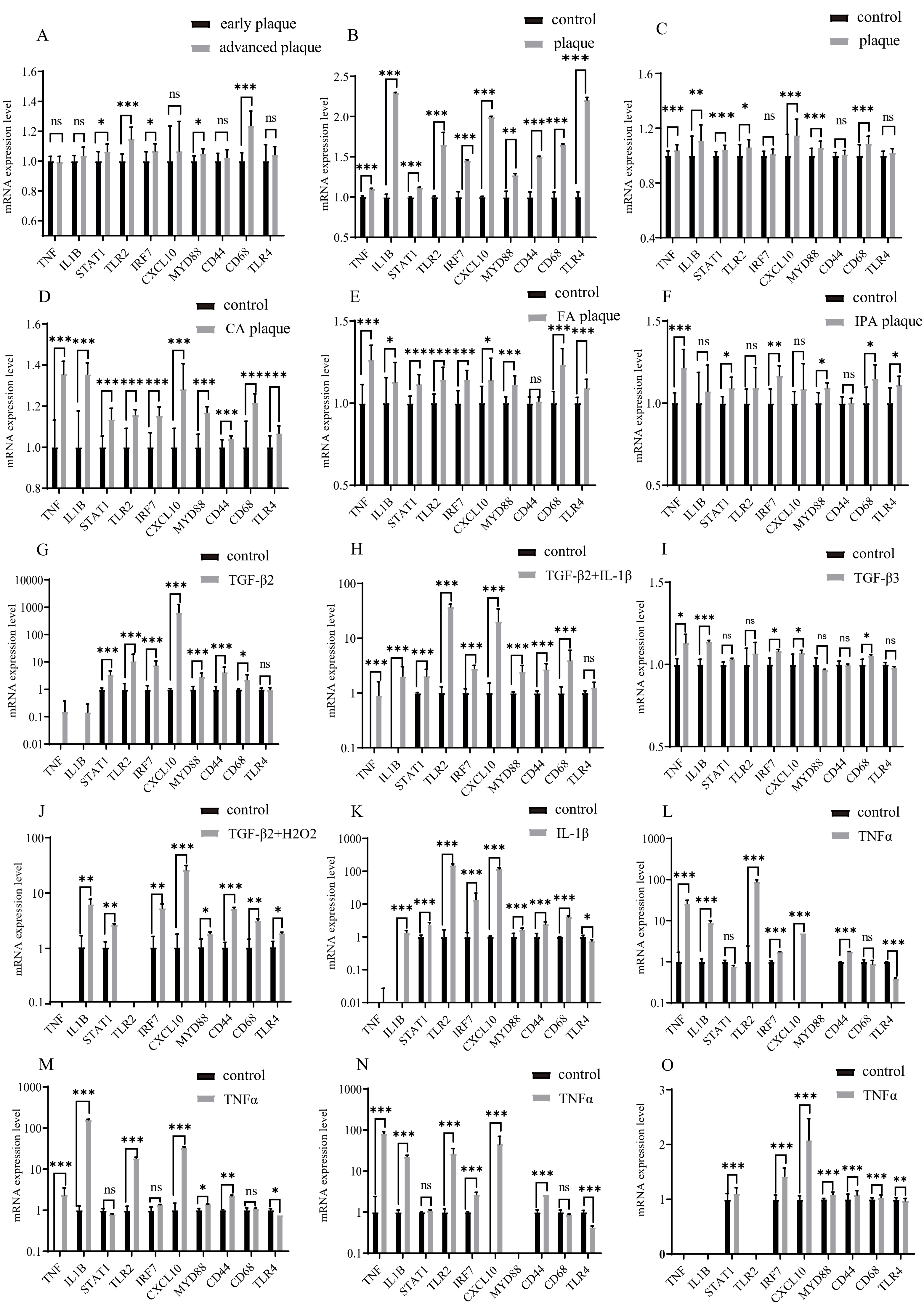

A
early plaque
advanced plaque
B
control
plaque
C
control
plaque
D
control
CA plaque
E
control
FA plaque
F
control
IPA plaque
G
control
TGF-β2
H
control
TGF-β2+IL-1β
I
control
TGF-β3
J
control
TGF-β2+H2O2
K
control
IL-1β
L
control
TNFα
M
control
TNFα
N
control
TNFα
O
control
TNFα
mRNA expression level
TNF
IL1B
STAT1
TLR2
IRF7
CXCL10
MYD88
CD44
CD68
TLR4
